# Two-step tandem synthesis of sugar-containing pyrimidine derivatives catalyzed by Lipozyme® TL IM in continuous-flow microreactors†

**DOI:** 10.1039/d4ra07120d

**Published:** 2024-12-02

**Authors:** Han-Jia Xie, Wen-Xuan Shao, Li-Hua Du, Ao-Ying Zhang, Hang Lin, Zong-Hao Huang, Guo-Neng Fu, Jia-Hong Shen, Bing-Lin Yan, Miao-Miao Xue, Lin Wang, Xi-Ping Luo

**Affiliations:** a College of Pharmaceutical Science, Zhejiang University of Technology Hangzhou 310014 Zhejiang China orgdlh@zjut.edu.cn +86 571 88320903 +86-189-690-693-99; b Zhejiang Hisun Pharmaceutical Co., Ltd. Taizhou 318000 Zhejiang China jhshen@hisunpharm.com; c Zhejiang Provincial Key Laboratory of Chemical Utilization of Forestry Biomass, Zhejiang A&F University Hangzhou 311300 Zhejiang China luoxiping@zafu.edu.cn

## Abstract

A novel series of sugar-containing pyrimidine derivatives have been synthesized *via* an effective and convenient two-step tandem synthesis in continuous-flow microreactors with excellent regioselectivity. Using continuous-flow microreactors to assemble two-step reactions into one unit can effectively avoid separating and purifying intermediates. Moreover, this method allows the optimization of reaction parameters for each unit individually. The salient features of this method include a reduction in the use of DMSO and mild reaction conditions (30–40 °C). Under the optimum reaction conditions, we can obtain the desired yield (34.8–69.1%) in a shorter time (40 min) than the shaking condition (48 h).

## Abstract 2

In continuous-flow microreactors, a two-step tandem scheme was developed to synthesize a series of sugar-containing pyrimidine derivatives with excellent regioselectivity, which can effectively avoid the separation and purification of intermediates.

## Graphical abstract

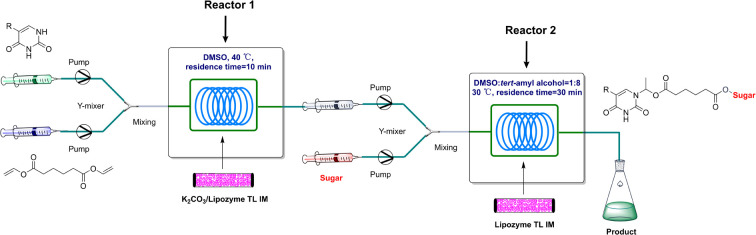

## Introduction

**Fig. 1:**
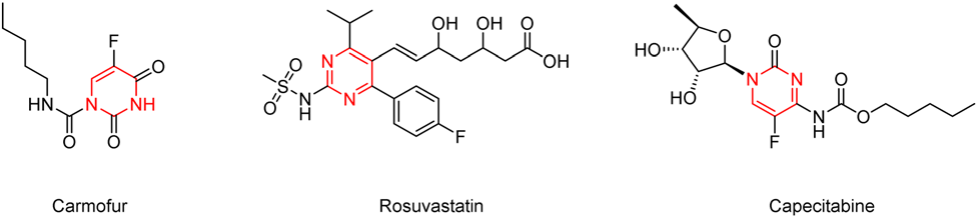
Drugs containing pyrimidine structural unit.

Pyrimidine structural motif is a favoured pharmacophore in many bioactive natural products and synthetic pharmaceutical drugs, such as rosuvastatin,^1^ carmofur,^2^ and capecitabine^3^ (Fig. 1). It demonstrates a wide range of exciting bioactivities, including anticancer, anticholesterol, and antibacterial effects.^4–10^ 5-Fluorouracil (5-Fu) is an antimetabolic agent whose action involves the irreversible inhibition of thymidylate synthase *via* competitive binding.^11,12^ However, it suffers from a short half-life, low bioavailability, and uncontrolled release. Moreover, both poor selectivity for cancer cells and a high incidence of normal tissue toxicity limit its therapeutic utility.^13^ The preparation of pyrimidine derivatives with highly targeted, high bioavailability, good lipid-soluble, and moderate half-life is a hot topic in medicinal chemistry. Researchers have synthesized many pyrimidine derivatives through modifications of 5-fluorouracil with a series of compounds such as small-molecule sugars, amino acids, short peptides, triazoles, quinolines, and porphyrin to improve the anticancer action and antitumor curative efficacy.^14–19^ Among these, saccharides occupy a unique and distinct place in organic chemistry. A significant number of drugs in use today rely on carbohydrates for part of their therapeutic action. Saccharides are also particularly effective for improving the water-solubility and dissolution behavior of parental drugs. Studies have shown that the structural modification of 5-fluorouracil by sugar compounds can synthesize more active and metabolically stable sugar-containing pyrimidine derivatives.^20^

In recent decades, numerous sugar-containing derivatives have been synthesized to improve their physicochemical, biopharmaceutical, and pharmacokinetic properties.^21^ The synthesis of these compounds is usually carried out by chemical or enzymatic methods.^22–24^ The traditional chemical synthesis of sugar-containing compounds usually requires “protection” or “protection–deprotection” steps, which increases the complexity of the reaction. Compared to the traditional way, enzymes can perform various transformations, including kinetic resolution, esterification, hydrolysis, reductions, oxidations, cyclization, aziridinations, and nitration reactions.^25–34^ The reactions catalyzed by enzymes are relatively mild and green, but the reaction time is usually long (∼24 h or longer). For example, Q. Wu *et al.*^35^ synthesized a series of monosaccharide-containing derivatives catalyzed by one enzyme in one pot; the enzymatic acylation of d-galactose occurred on C2–OH and was attributed to the regioselectivity of the enzyme, with a yield of 13–43% at 50 °C for 48 h. In the last decades, continuous-flow microreactors combined with enzymes have become an efficient way to reduce the reaction time and get a higher yield.^36–38^

**Scheme 1:**
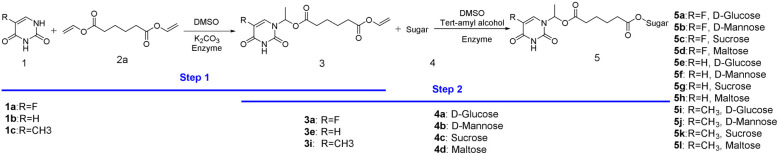
Synthesis of sugar-containing pyrimidine compounds in continuous-flow microreactors.

Continuous manufacturing has attracted the attention from both industry and academia due to improvements in mass and heat transfer, significant process intensification, ability to operate 24/7, and easier optimization through adjustments of simple parameters such as flow, pressure, and temperature.^39–41^ Compared to batch reactions, continuous flow presents significant advantages, including a high ratio of surface-to-volume, enhanced heat transfer and precise temperature control, higher reaction rates, good control of the residence time, easy automation and scale-up, and cost-effectiveness.^42–44^ The processes in the reactor can be enhanced by miniaturizing the reactor, including intensified mass transfer, better control of reaction parameters, and efficient reaction regioselectivity.^44–47^ Microfluidics is a viable alternative to the time and energy-consuming conventional synthesis processes, as significantly faster reaction rates can be achieved compared to the traditional batch processes.^48–50^ In addition, continuous flow microreactors have proven to be highly effective in materials preparation, consistently delivering outstanding results.^51–55^ Recent studies have demonstrated that the use of a constant flow microreactor provides a valuable platform for the development of multistep syntheses by integrating multiple unit operations into one single network and avoiding a lengthy isolation process and purification of the intermediates.^56–60^ On the basis of previous research in our group, the main objective of this work was to establish an efficient two-step tandem method for the synthesis of sugar-containing pyrimidine compounds in continuous-flow microreactors (Scheme 1). The first preliminary batch experiments were performed for each step. In the first step, considering the poor solubility of 5-fluorouracil, 5-fluorouracil and divinyl adipate were dissolved in DMSO, and then a catalyst was added. The reactants were mixed and reacted by shaking 180 revolutions per min (rpm) at 50 °C for 24 h, 83% product was obtained with the mixture of 12% K_2_CO_3_/Lipozyme® TL IM as catalyst (K_2_CO_3_ accounts for 12% of the total catalyst mass). Considering the solubility of sugar, we screened several solvents in the next step (Table 1). Using d-glucose as a substrate, no product was formed with pyridine or DMSO as solvent (entry 2 and 4 in Table 1). Although there were good yields in THF and acetone, the poor solubility of sugar in those solvents will cause blockage of the reaction pipeline. According to Table 1, the reaction proceeds well with Lipozyme® TL IM as catalyst and DMSO : *tert*-amyl alcohol = 1 : 8 as solvent.

**Table 1:** The effect of reaction media on the enzymatic synthesis of sugar-containing pyrimidine derivatives in shaker reactors^a^

Entry	Solvent	Temperature	Yield^b^ (%)
1	THF	50 °C	58%
2	Pyridine	50 °C	n.d.
3	Acetone	50 °C	62%
4	DMSO	50 °C	n.d.
5	DMSO/*tert*-amyl alcohol = 1 : 1	50 °C	n.d.
6	DMSO/*tert*-amyl alcohol = 1 : 2	50 °C	n.d.
7	DMSO/*tert*-amyl alcohol = 1 : 4	50 °C	Trace
8	DMSO/*tert*-amyl alcohol = 1 : 6	50 °C	18%
9	DMSO/*tert*-amyl alcohol = 1 : 8	50 °C	53%
10	DMSO/*tert*-amyl alcohol = 1 : 10	50 °C	55%

a General experimental conditions: in the shaker reactors, the second step was a synthesis of sugar-containing pyrimidine derivatives using 1-(1-(5-fluorouracil))-ethyl vinyl adipate (3a) and d-glucose (4a) as a probe reaction. In the preliminary exploration experiments, the substrate molar ratio of 1-(1-(5-fluorouracil))-ethyl vinyl adipate (3a) and d-glucose is 2 : 1, enzyme 870 mg, 180 rpm, 50 °C, 24 h.

b Isolated yield. Yield: 100 × (actual received amount/ideal calculated amount).

## Results and discussion

### The effect of reaction parameters on the synthesis of pyrimidine vinyl ester intermediates

Encouraged by these results, we were keen to develop a two-step tandem continuous flow protocol for this reaction. The flow chemistry implementation of the synthesis of pyrimidine derivatives in a mild, two-step sequence could separate this transformation, allowing the application of different conditions. Each of the two-unit operations (Markovnikov addition and transesterification) was studied and optimized before being assembled as a continuous network. For the first step, using 5-fluorouracil and divinyl adipate as a probe reaction, we discussed several reaction parameters, including substrate molar ratio, catalysts, reaction temperature, and residence time. From Table 2, we can find that with the increase of divinyl adipate (2a), the yield increased gradually. Considering the atomic economy, we chose 5-fluorouracil : divinyl adipate = 1 : 8 as the optimum substrate molar ratio. Based on the shaking research, the mixture of K_2_CO_3_/Lipozyme® TL IM with 14% K_2_CO_3_ mass fraction is the most effective catalyst. A stable yield of 88% product was obtained with the reaction running continuously for 10 min at 40 °C (Table 2).

**Table 2:** The effect of reaction parameters on the synthesis of pyrimidine vinyl ester intermediates in a continuous-flow microreactor^a^

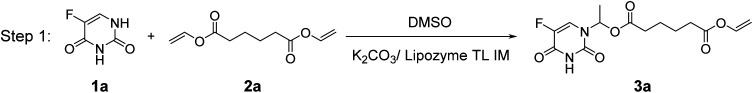					
Entry	Substrate molar ratio	Catalysts	Temperature	Time	Yield^b^ (%)
1	1a : 2a = 1 : 6	12% K_2_CO_3_/Lipozyme® TL IM	50 °C	30 min	43%
2	1a : 2a = 1 : 8	12% K_2_CO_3_/Lipozyme® TL IM	50 °C	30 min	60%
3	1a : 2a = 1 : 10	12% K_2_CO_3_/Lipozyme® TL IM	50 °C	30 min	62%
4	1a : 2a = 1 : 8	10% K_2_CO_3_/Lipozyme® TL IM	50 °C	30 min	57%
5	1a : 2a = 1 : 8	14% K_2_CO_3_/Lipozyme® TL IM	50 °C	30 min	79%
6	1a : 2a = 1 : 8	16% K_2_CO_3_/Lipozyme® TL IM	50 °C	30 min	70%
7	1a : 2a = 1 : 8	18% K_2_CO_3_/Lipozyme® TL IM	50 °C	30 min	61%
8	1a : 2a = 1 : 8	14% K_2_CO_3_/Lipozyme® TL IM	30 °C	30 min	73%
9	1a : 2a = 1 : 8	16% K_2_CO_3_/Lipozyme® TL IM	30 °C	30 min	60%
10	1a : 2a = 1 : 8	14% K_2_CO_3_/Lipozyme® TL IM	40 °C	30 min	84%
11	1a : 2a = 1 : 8	16% K_2_CO_3_/Lipozyme® TL IM	40 °C	30 min	77%
12	1a : 2a = 1 : 8	14% K_2_CO_3_/Lipozyme® TL IM	40 °C	40 min	65%
13	1a : 2a = 1 : 8	16% K_2_CO_3_/Lipozyme® TL IM	40 °C	10 min	81%
14	1a : 2a = 1 : 8	14% K_2_CO_3_/Lipozyme® TL IM	40 °C	10 min	88%
15	1a : 2a = 1 : 8	16% K_2_CO_3_/Lipozyme® TL IM	40 °C	20 min	79%
16	1a : 2a = 1 : 8	14% K_2_CO_3_/Lipozyme® TL IM	40 °C	20 min	89%

a General experimental conditions: in the continuous flow reactors, feed 1, 10 mL solvent contained 5.0 mmol 5-fluorouracil; feed 2, 10 mL solvent contained 40.0 mmol divinyl adipate, mixed catalyst 870 mg.

b Isolated yield. Yield: 100 × (actual received amount/ideal calculated amount).

### The effect of reaction parameters on the synthesis of sugar-containing pyrimidine derivatives

**Fig. 2:**
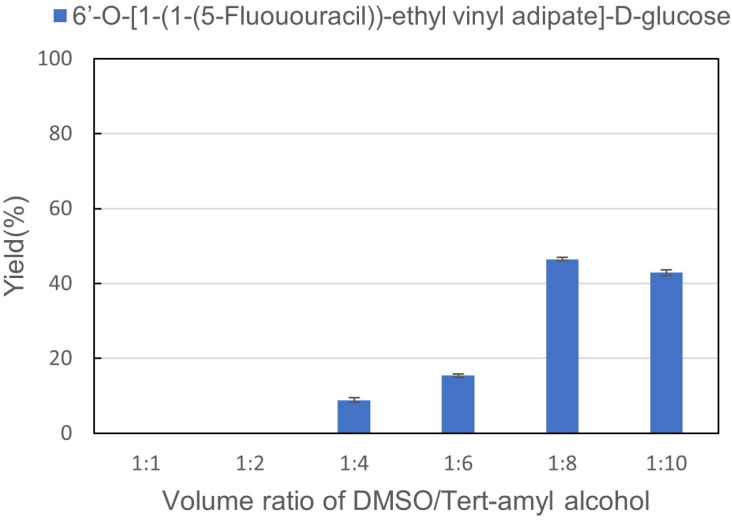
The effect of solvent volume ratio on the synthesis of sugar-containing pyrimidine derivatives in continuous-flow microreactors.

The second step was a synthesis of sugar-containing pyrimidine derivatives using 1-(1-(5-fluorouracil))-ethyl vinyl adipate (3a) and d-glucose (4a) as a probe reaction (Step 2). We also studied reaction parameters such as solvents, substrate molar ratio, reaction temperature, and residence time in continuous-flow microreactors. As a non-aqueous medium, organic solvents play an important role in the enzymatic synthesis of sugar-containing pyrimidine derivatives. Solvent mediators can directly or indirectly affect enzymes' properties by interacting with enzymes' essential water and changing the structure and flexibility of enzymatic proteins. In addition, organic solvents can also affect the solubility of substrates and products. Solid precipitation will cause blockage of the reaction pipeline and affect the yield in continuous-flow microreactors. Thus, the DMSO : *tert*-amyl alcohols = 1 : 1, 1 : 2, 1 : 4, 1 : 6, 1 : 8, 1 : 10 were studied, and the results are shown in Fig. 2. We can find that the yield increased when the volume of *tert*-amyl alcohols increased. Meanwhile, DMSO is also a key condition to ensure the full dissolution of substrates and products, which can effectively avoid blockage of the reaction pipeline. Therefore, we chose DMSO : *tert*-amyl alcohol = 1 : 8 as the optimum solvent for enzymatic synthesis of sugar-containing pyrimidine derivatives in continuous-flow microreactors.

**Fig. 3:**
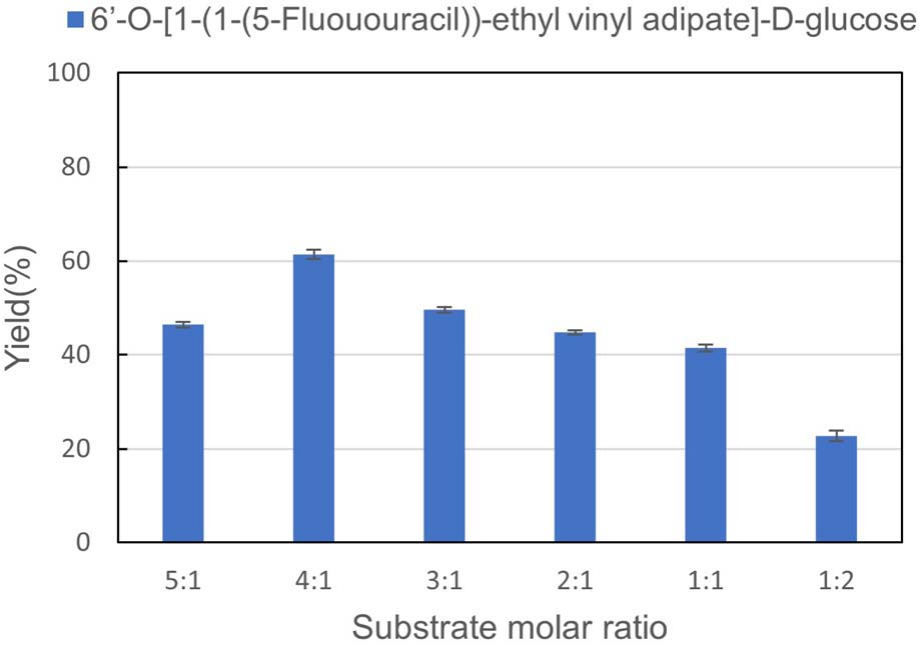
The effect of substrate molar ratio on the synthesis of sugar-containing pyrimidine derivatives in continuous-flow microreactors.

Enzymes react with specific structures according to their spatial structure. Changing the molar ratio of substrates will affect the catalytic efficiency of the enzyme. The molar ratios of 1-(1-(5-fluorouracil))-ethyl vinyl adipate : d-glucose = 5 : 1, 4 : 1, 3 : 1, 2 : 1, 1 : 1, 1 : 2 were investigated, catalyzing by Lipozyme® TL IM under 50 °C. The results can be seen in Fig. 3. It can be found that the yield was not satisfactory when the amount of d-glucose was higher. With the increase of 1-(1-(5-fluorouracil))-ethyl vinyl adipate, the yield increases gradually. When the molar ratio of 1-(1-(5-fluorouracil))-ethyl vinyl adipate : d-glucose = 4 : 1, the reaction reached equilibrium. Therefore, the molar ratio of 1-(1-(5-fluorouracil))-ethyl vinyl adipate : d-glucose = 4 : 1 was the best substrate molar ratio for synthesizing sugar-containing pyrimidine derivatives in microreactors.

**Fig. 4:**
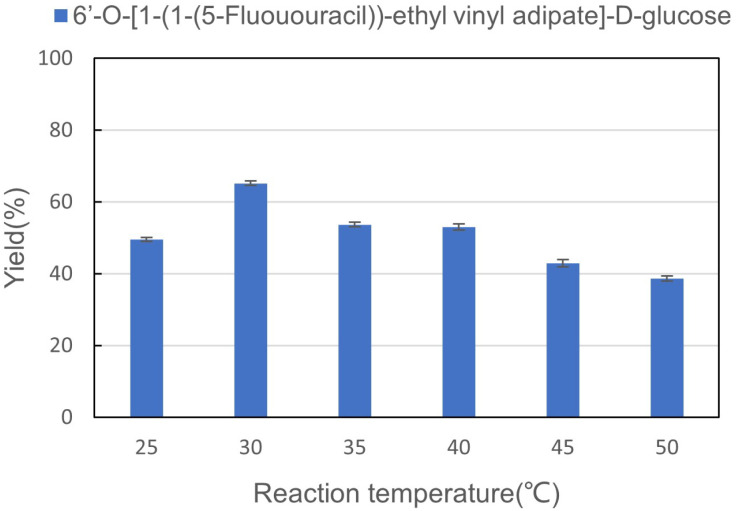
The effect of reaction temperature on the synthesis of sugar-containing pyrimidine derivatives in continuous-flow microreactors.

Reaction temperature significantly influences the activity of enzymes, which are proteins or RNA molecules with catalytic properties. When the temperature is too high or too low, the catalytic activity of the enzymes is inhibited. It is of great significance to explore the effect of reaction temperature on the enzymatic synthesis of sugar-containing pyrimidine derivatives in microreactors. The reaction was carried out in a microreactor for 30 min. As shown in Fig. 4, the optimal yield of the enzymatic reaction was obtained at 30 °C. When the temperature was lower than 30 °C, the catalytic activity of the enzyme decreased, resulting in a decrease in the yield, while the reaction was obviously inhibited when the temperature reached 50 °C. Therefore, we chose 30 °C as the best reaction temperature for the synthesis of sugar-containing pyrimidine derivatives in microreactors.

**Fig. 5:**
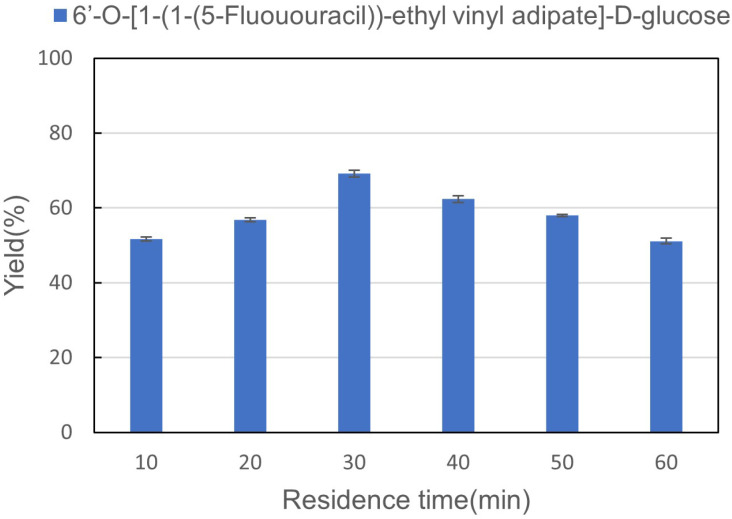
The effect of residence time on the synthesis of sugar-containing pyrimidine derivatives in continuous-flow microreactors.

The residence time has an important influence because the enzymatic reaction is reversible, so we studied the influence of reaction time on the synthesis of sugar-containing pyrimidine derivatives in microreactors. The investigated residence time was 10 min, 20 min, 30 min, 40 min, 50 min, and 60 min. From Fig. 5, when the residence retention time is 10 minutes, 51.7% yield can be obtained. With the increase in residence time, the reaction obtained the best result when reacted for 30 min. The reaction yield decreased with the increase in residence time. Therefore, 30 min was chosen as the optimal reaction time for enzymatic synthesis of sugar-containing pyrimidine derivatives in microreactors.

### Two-step tandem synthesis of sugar-containing pyrimidine derivatives

With the optimum reaction conditions in hand, we develop a two-step tandem continuous-flow protocol for the synthesis of sugar-containing pyrimidine derivatives. Under the optimum reaction conditions, we synthesized a series of pyrimidine vinyl ester intermediates and sugar-containing pyrimidine derivatives. Compared with the shaking reaction, the two-step tandem continuous-flow protocol can eliminate the need for purification and isolation of intermediates. Meanwhile, using this method can obtain the desired yield (34.8–69.1%) in a shorter time (40 min) than the shaking condition (48 h). From Table 3, we can find that the yield of monosaccharides is higher than the disaccharides. The products were detected by ^13^C NMR, and ^1^H NMR.

**Table 3:** Comparison of continuous flow reactors and shaker reactors for the two-step synthesis of sugar-containing pyrimidine derivatives

Entry	Sugar	Product	Method^a^	Time	Yield^b^ (%)
1	d-Glucose	5a	A	40 min	69.1 ± 0.9
			B	48 h	54.4 ± 1.2
2	d-Mannose	5b	A	40 min	52.7 ± 0.6
			B	48 h	46.3 ± 0.9
3	d-Sucrose	5c	A	40 min	34.8 ± 1.1
			B	48 h	32.9 ± 0.9
4	d-Maltose	5d	A	40 min	36.1 ± 0.8
			B	48 h	32.5 ± 0.5
5	d-Glucose	5e	A	40 min	52.7 ± 0.3
			B	48 h	38.9 ± 0.6
6	d-Mannose	5f	A	40 min	55.4 ± 1.1
			B	48 h	42.7 ± 1.4
7	d-Sucrose	5g	A	40 min	37.2 ± 1.6
			B	48 h	29.9 ± 1.9
8	d-Maltose	5h	A	40 min	36.3 ± 0.4
			B	48 h	33.6 ± 0.8
9	d-Glucose	5i	A	40 min	51.8 ± 1.7
			B	48 h	42.4 ± 1.9
10	d-Mannose	5j	A	40 min	50.3 ± 1.1
			B	48 h	45.2 ± 0.9
11	d-Sucrose	5k	A	40 min	35.9 ± 0.8
			B	48 h	31.6 ± 1.2
12	d-Maltose	5l	A	40 min	38.3 ± 1.3
			B	48 h	32.9 ± 1.9

a General experimental conditions: Method A: continuous flow reactors, step 1: feed 1, dissolve 5 mmol of pyrimidine analogs in 10 mL DMSO; feed 2, 40 mmol of diethylene-adipate was taken and added to DMSO to prepare 10 mL of solution, residence time 10 min, mixed catalyst 870 mg, 40 °C. Step 2: feed 3, 2.22 mL above reaction solution mixed with 17.88 mL *tert*-amyl alcohol; feed 4, sugar (0.28 mmol) was dissolved in 2.22 mL DMSO and 17.78 mL *tert*-amyl alcohol, residence time 30 min, enzyme 870 mg, 30 °C. Method B: shaker reactors, step 1: add 5 mmol of pyrimidine analogs, 40 mmol of diethylene-adipate and 20 mL DMSO to a 50 mL Erlenmeyer flask, mixed catalyst 870 mg, 180 rpm, 50 °C, 24 h. Step 2: add 1 mmol of pyrimidine vinyl ester intermediates, 0.25 mmol of sugar and 20 mL mixed solvent (DMSO : *tert*-amyl alcohol = 8 : 1) to a 50 mL Erlenmeyer flask, enzyme 870 mg, 180 rpm, 50 °C, 24 h.

b Isolated yield. Yield: 100 × (actual received amount/ideal calculated amount). The data are presented as average ± standard deviation (SD) of triplicate experiments.

## Experimental section

**Fig. 6:**
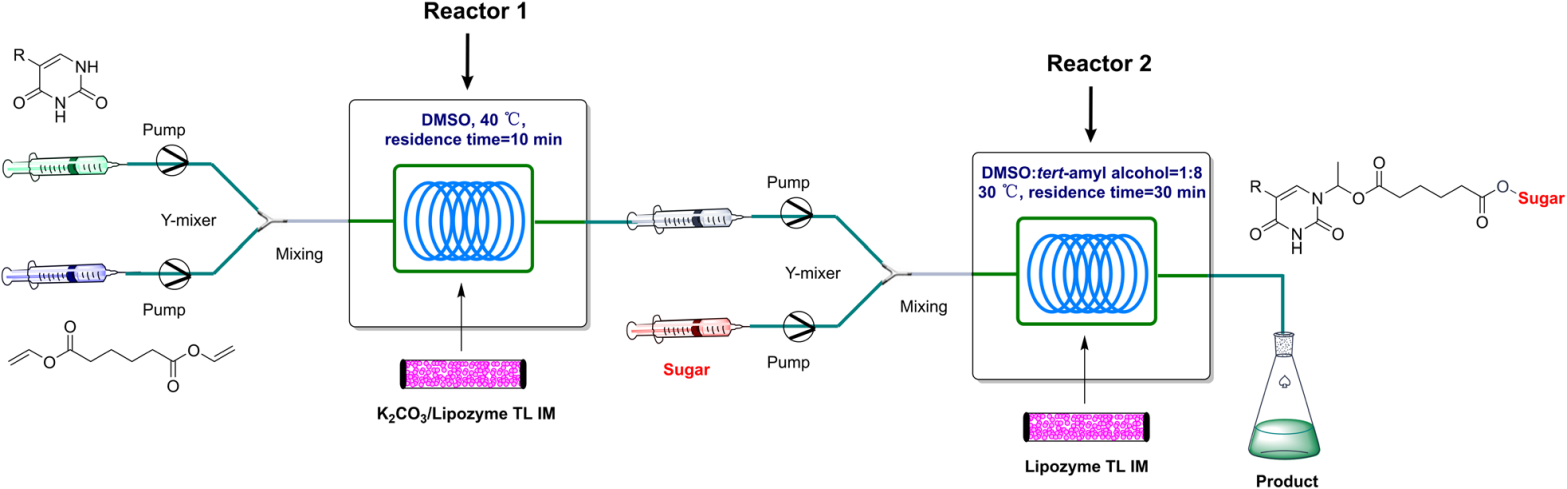
The experimental setup of two-step tandem synthesis of sugar-containing pyrimidine derivatives in continuous-flow microreactors.

With the optimum reaction conditions in hand, we develop a two-step tandem continuous-flow protocol for the synthesis of sugar-containing pyrimidine derivatives (Fig. 6). The experimental setup consisted of two micro-system devices: two syringe pumps, coil reactor 1 and coil reactor 2, and Y-shaped mixers (*φ* = 1.8 mm). Syringe pumps (Harvard apparatus PHD 2000) were used to introduce separate feed streams to PFA coil reactors (2.0 mm I.D.). In the first microreactor (Reactor 1), the solution of the 5-fluorouracil (1a) comes into contact with the solution of divinyl adipate (2a), resulting in the formation of the pyrimidine vinyl ester intermediates (3a). This intermediate is transformed by the addition of d-glucose (4a) to yield the desired sugar-containing pyrimidine derivative (5) in the second microreactor (Reactor 2). Coil reactor 1 was filled with K_2_CO_3_/Lipozyme® TL IM (catalyst reactivity: 250 IUN g^−1^), and coil reactor 2 was filled with Lipozyme® TL IM, both submerged into a thermostatic water bath to control the reaction temperature. The resulting stream was connected to a sample vial to collect the final mixture. The main products were separated by silica gel chromatography and were confirmed by ^1^H NMR, and ^13^C NMR.

## Conclusion

In summary, the two-step tandem continuous-flow protocol described herein can obtain target compounds mildly and efficiently. By telescoping the Markovnikov addition and esterification steps into a single, continuous, and uninterrupted reactor network, thereby circumventing the need to isolate and purify the intermediate product. The scope of the reaction was tested by varying the pyrimidine derivatives, vinyl esters, and sugars. The major features of this method include a reduction in the use of DMSO, mild reaction condition (40 °C for the first step and 30 °C for the second step), shorter reaction time (10 min for the first step and 30 min for the second step), using an enzyme as a catalyst resulting in high regioselectivities. We can use this technique to quickly synthesize the related compound library for the next screening of drug activity.

## Data availability

The authors confirm that the data supporting the findings of this study are available within its ESI.†

## Conflicts of interest

There are no conflicts to declare.

## Supplementary Material

RA-014-D4RA07120D-s001
