# Supplementary material for: Two-step tandem synthesis of sugar-containing pyrimidine derivatives catalyzed by Lipozyme® TL IM in continuous-flow microreactors
Source: RSC Adv. 2024 Dec 2;14(51):38193–9. doi: 10.1039/d4ra07120d (PMC11610444; doi:10.1039/d4ra07120d)
Supplement: RA-014-D4RA07120D-s001 [file RA-014-D4RA07120D-s001.pdf]

Supplementary Information (SI) for RSC Advances.
This journal is © The Royal Society of Chemistry 2024

Supporting Information for

Two-step Tandem Synthesis of Sugar-containing Pyrimidine Derivatives Catalyzed by Lipozyme® TL IM in Continuous-Flow Microreactors

Han-Jia Xie, [#a] Wen-Xuan Shao, [#a] Li-Hua Du, *[a] Ao-Ying Zhang, [a] Hang Lin, [a] Zong-Hao Huang, [a] Guo-Neng Fu, [a] Jia-Hong Shen, [b] Bing-Lin Yan, [a] Miao-Miao Xue, [a] Lin Wang, [a] and Xi-Ping Luo *[c]

Materials

Unless otherwise stated, all chemicals were obtained from commercial sources and used without further purification. Lipozyme® TL IM from *Thermomyces lanuginosus* was purchased from Novo Nordisk (Copenhagen, Denmark). Uracil, 5-fluorouracil, 5-methyluracil, and divinyl adipate were purchased from Energy Chemical. D-glucose, D-mannose, Sucrose, and Maltose were purchased from China Pharmaceutical Group Chemical Reagents Co., Ltd. Harvard Apparatus PHD 2000 was purchased from Harvard University (Holliston, Massachusetts, USA). The flow reactor and Y-mixer were purchased from Beijing Haigui Medical Engineering Design Co., Ltd (Beijing China). A 400 MHz NMR spectrometer (Billerica, MA, USA) was also used in this study.

Purification of the product

When the conversion of the sugar-containing pyrimidine derivatives reaches a maximum (determined by TLC), the reaction is terminated by filtering the enzyme. Since the reaction took a mixture of *tert*-amyl alcohol/DMSO as solvent, the reaction solution was first distilled under reduced pressure to remove the *tert*-amyl alcohol from the reaction solution and then the remaining DMSO was extracted. Ice water and ethyl acetate were used as solvents for the extraction of the reaction solution. The product is separated by silica gel chromatography (mobile phase dichloromethane/methanol, 10/1.5). Purification was monitored by TLC. The graded fractions containing the major product were combined, the solvent evaporated and the residue was analyzed by ^{1}H NMR, ^{13}C NMR.

Experimental setup

A two-step tandem continuous-flow protocol for the synthesis of sugar-containing pyrimidine derivatives is described in Fig. 1. The experimental setup was consisted of two micro-system devices: two syringe pumps, coil reactor 1 and coil reactor 2, Y-shaped mixers (ϕ= 1.8 mm). Syringe pumps (Harvard apparatus PHD 2000) were used to introduce separate feed streams to 3.1 m PFA coil reactors (2.0 mm I.D.). In the first microreactor (Reactor 1) the solution of pyrimidine analogs (5-fluorouracil, 5-methyluracil, uracil) come into contact with the solution of divinyl adipate, resulting in the formation of the pyrimidine vinyl ester intermediates. This intermediate is transformed by the addition of sugars to yield the desired sugar-containing pyrimidine derivatives in the second microreactor (Reactor 2). Coil reactor 1 was filled with K_2CO_3/ Lipozyme® TL IM (catalyst reactivity: 250 IUN g^{-1}) and coil reactor 2 was filled with Lipozyme® TL IM, both submerged into a thermostatic water bath to control the reaction temperature. The final products stream exiting this unit is directed into collection bottles.

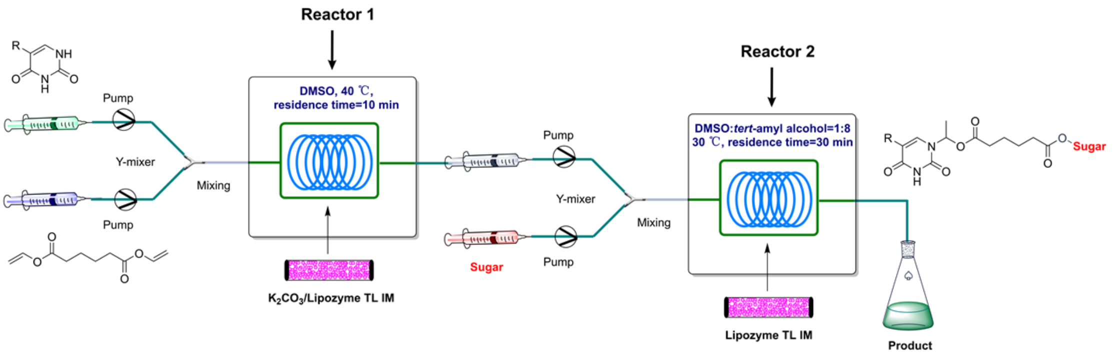

Fig. 1 The experimental setup of two-step tandem synthesis of sugar-containing pyrimidine derivatives in microreactors

The Space-time yield (STY) in the continuous-flow Microreactors and the Shaker Reactors

Table 1. Enzymatic Synthesis in the Continuous-Flow Microreactor and the Shaker Reactor.

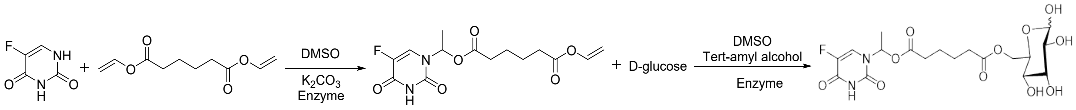

Entry	Reactor	STY (g L^{-1} h^{-1})	Yield (%)
1	Continuous-Flow Microreactor	6.85	69.1±0.9
2	Shaker Reactor	1.86×10^{-3}	54.4±1.2

Experiment

General Procedure for Sugar-containing Pyrimidine Derivatives Synthesis in Continuous Flow Microreactors.

Method A (Reaction method under microfluidics):

5 mmol of pyrimidine analogs (5-fluorouracil, 5-methyluracil, uracil) were taken and added to 10 mL of DMSO solution and dissolved to obtain a DMSO solution of 0.5 mmol mL^{-1} of pyrimidine analogs (feed 1); 40 mmol of diethylene-adipate was taken and added to DMSO to prepare 10 mL of solution (feed 2). A mixture of 0.87 g of catalyst (0.1218 g of K_2CO_3 and 0.7482 g of Lipozyme® TL IM) was evenly filled in the reaction tube. Feeds 1 and 2 were placed in separate 10 mL feeders and mixed together in a Y-mixer. The residence time was 10 min and the reaction temperature was controlled by a water bath thermostat at 40 °C.

At the end of the reaction, 2.22 mL of the above reaction solution was slowly added dropwise to 17.78 mL of *tert*-amyl alcohol and mixed well (feed 3). 0.28 mmol of sugar compounds (D-glucose, D-mannose, sucrose, maltose) was added to 2.22 mL of DMSO solution and dissolved thoroughly, then 17.78 mL of *tert*-amyl alcohol was added and filled into the syringe (feed 4). The reaction solution in the two feeds was mixed at a flow rate of 10.4 μL min^{-1} by a microfluidic pump through a Y-mixer and then fed into the reaction channel at 30 °C. The resulting stream (20.8 μL min^{-1}) was connected to a sample vial for collection of the final mixture. The reaction was followed up qualitatively by thin layer chromatography TLC. After the reaction was completed, the reactants were post-treated and the pure compounds obtained were subjected to structural characterization. As the reaction took a mixture of *tert*-amyl alcohol/ DMSO as the solvent, the reaction solution was first distilled under reduced pressure to remove the *tert*-amyl alcohol from the reaction solution, and then the remaining DMSO was extracted. The extraction was carried out using ice water and ethyl acetate as solvents. The silica gel used for the column chromatography was 300-400 mesh, wet loaded and purified using dichloromethane: methanol = 10:1.5 as eluent.

General Procedure for Sugar-containing Pyrimidine Derivatives Synthesis in Shaker Reactor.

Method B (Reaction method under shaking conditions):

5 mmol of pyrimidine compounds (5-fluorouracil, 5-methyluracil, uracil) was placed in a 50 mL conical flask and dissolved by adding 20 mL of DMSO. This was followed by the addition of 40 mmol of the vinyl ester compound, diethylene-adipate, which was shaken until mixed, and 0.87 g of mixed catalyst (a mixture of 0.1218 g K_2CO_3 and 0.7482 g Lipozyme® TL IM) was added. The conical flask was placed in a water bath thermostatic shaking reactor at 50 °C and 180 r.p.m. and the reaction was followed qualitatively by TLC.

1 mmol of pyrimidine vinyl ester intermediates (1-(1-(5-fluorouracil)-ethyl vinyl adipate, 1-(1-(5-methyl-uracil)-ethyl vinyl adipate, 1-(1-uracil)-ethyl vinyl adipate) were placed in a 50 mL conical flask and dissolved by adding 2.22 mL of DMSO, 17.78 mL of *tert*-amyl alcohol, followed by the addition of 0.25 mmol of the sugars. The reaction was monitored qualitatively by TLC for 24 h at 50 °C and 180 r.p.m. in a water bath with a thermostatic shaker. At the end of the reaction, the reactants were post-treated and the resulting pure compounds were subjected to structural characterization. Since the reaction took a mixture of *tert*-amyl alcohol/DMSO as solvent, the reaction solution was first distilled under reduced pressure to remove the *tert*-amyl alcohol from the reaction solution and then the remaining DMSO was extracted. Ice water and ethyl acetate were used as solvents for the extraction of the reaction solution. The silica gel used for the column chromatography was 300-400 mesh, wet loaded and purified using dichloromethane: methanol = 10:1.5 as eluent.

Thin Layer Chromatography

Analytical TLC was performed on silica gel 60 plates (Merck) using dichloromethane/methanol (10:1.5, v/v) as the developing solvent. Spots were detected by UV irradiation at 254 nm.

Characterization data for products

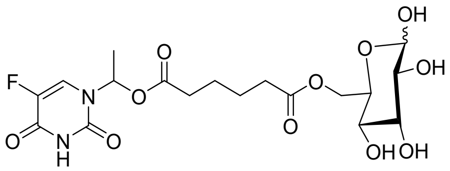

6'-O-[1-(1-(5-Fluououracil))-ethyl vinyl adipate]-D-glucose (5a): white solid, isolated yield 69.1%; ^{1}H NMR (500 MHz, DMSO-d_6): δ 11.89 (s, 1H, 3-NH), 8.18 (dd, *J*=6.9, 1.5 Hz, 1H, 6-H), 6.77 (qd, *J*=6.3, 1.6 Hz, 1H, N-C<u>H</u>(CH$_3$)-O), 6.66 (d, *J*= 6.2 Hz, 0.5H, C1'-OH of β-D-glucose), 6.34 (d, *J*=4.4 Hz, 0.5H, C1'-OH of α-D-glucose), 5.10 (s, 0.5H, C1'-H of β-D-glucose), 5.04 (s, 0.5H, C1'-H of α-D-glucose), 5.00-4.84 (m, 1.5H, C4'-OH, C2'-OH of β-D-glucose, C4'-OH of α-D-glucose), 4.75 (s, 0.5H, C2'-OH of α-D-glucose), 4.53 (s, 0.5H, C3'-OH of α-D-glucose), 4.28 (ddt, *J*=18.1, 11.7, 2.0 Hz, 1.5H, C6'-Ha, C3'-OH of β-D-glucose, C6'-Ha of α-D-glucose), 3.99 (tdd, *J*=11.8, 6.5, 1.5 Hz, 1H, C6'-Hb of β-D-glucose, C6'-Hb of α-D-glucose), 3.77 (ddd, *J*=10.0, 6.2, 2.0 Hz, 0.5H, C5'-H of α-D-glucose), 3.43 (t, *J*=9.1 Hz, 0.5H, C5'-H of β-D-glucose), 3.33-3.30 (m, 0.5H, C3'-H of α-D-glucose), 3.13 (d, *J*=9.0 Hz, 1H, C2'-H of β-D-glucose, C2'-H of α-D-glucose), 3.04 (td, *J*=9.3, 6.5 Hz, 1H, C3'-H of β-D-glucose, C4'-H of α-D-glucose), 2.91 (t, *J*=8.5 Hz, 0.5H, C4'-H of β-D-glucose), 2.39-2.26 (m, 4H, -CH$_2$-CH$_2$-), 1.54 (t, *J*=5.9 Hz, 7H, -CH(C<u>H$_3$</u>)-O-C=O-C<u>H$_2$</u>-, -C<u>H$_2$</u>-C=O-O-). ^{13}C NMR (126 MHz, CDCl$_3$): δ 172.69 (C-8), 171.09 (C-13), 157.07, 156.86 (C-4), 148.42 (C-2), 141.17, 139.33 (C-5), 124.98, 124.71 (C-6), 96.88 (C-1' of β-D-glucose), 92.27 (C-1' of α-D-glucose), 76.40 (C-3' of β-D-glucose), 75.14 (C-7), 74.69 (C-2' of β-D-glucose), 73.48 (C-5' of β-D-glucose), 72.87 (C-3' of α-D-glucose), 72.18 (C-2' of α-D-glucose), 70.57 (C-4' of α-D-glucose), 70.17 (C-4' of β-D-glucose), 69.12 (C-5' of

α-D-glucose), 63.95 (C-6′), 33.00, 32.95, 32.82 (C-9, C-12), 23.69, 23.50 (C-10, C-11), 18.43 (C-14). HRMS: $C_{18}H_{25}FN_2NaO_{11}$ for $[M+Na]^+$, calculated 487.1335, found 487.1346.

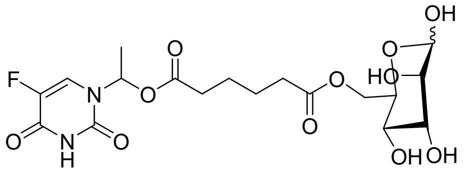

6′-O-[1-(1-(5-Fluououracil))-ethyl vinyl adipate]-D-mannose (5b): white solid, isolated yield 52.7%; ^{1}H NMR (500 MHz, DMSO-d_6): δ 11.89 (s, 1H, 3-NH), 8.19 (d, *J*=6.9 Hz, 1H, 6-H), 6.77 (qd, *J*=6.2, 1.5 Hz, 1H, N-C$\underline{H}$(CH_3)-O), 6.36 (d, *J*=4.5 Hz, 1H, C1′-OH of D-Mannose), 4.86 (t, *J*=5.5 Hz, 2H, C1′-H, C4′-OH of D-Mannose), 4.61 (d, *J*=4.1 Hz, 1H, C3′-OH of D-Mannose), 4.54 (d, *J*=7.5 Hz, 1H, C2′-OH of D-Mannose), 4.30 (dd, *J*=11.6, 2.0 Hz, 1H, C6′-H of D-Mannose), 4.01 (dd, *J*=11.6, 6.8 Hz, 1H, C6′-H of D-Mannose), 3.71 (ddd, *J*=9.1, 6.8, 1.9 Hz, 1H, C5′-H of D-Mannose), 3.57-3.50 (m, 2H, C2′-H, C3′-H of D-Mannose), 3.39 (dd, *J*=9.5, 5.1 Hz, 1H, C4′-H of D-Mannose), 2.39-2.27 (m, 4H, -C$\underline{H_2}$-C$\underline{H_2}$-), 1.54 (dd, *J*=7.9, 4.8 Hz, 7H, -CH(C$\underline{H_3}$)-O-C=O-C$\underline{H_2}$-, -C$\underline{H_2}$-C=O-O-). ^{13}C NMR (126 MHz, DMSO-d_6): δ 173.21 (C-8), 171.58 (C-13), 157.55, 157.34 (C-4), 148.89 (C-2), 141.64, 139.80 (C-5), 125.47, 125.19 (C-6), 94.52 (C-1′ of D-Mannose), 75.61 (C-2′ of D-Mannose), 71.76(C-3′ of D-Mannose), 70.84(C-5′ of D-Mannose), 67.61(C-4′ of D-Mannose), 64.68(C-6′ of D-Mannose), 33.51, 33.30 (C-9, C-12), 24.16, 23.99 (C-10, C-11), 18.90 (C-14). HRMS: $C_{18}H_{25}FN_2NaO_{11}$ for $[M+Na]^+$, calculated 487.1335, found 487.1341.

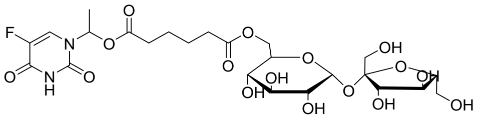

6′-O-[1-(1-(5-Fluououracil))-ethyl vinyl adipate]-sucrose (5c): white solid, isolated yield 34.8%; ^{1}H NMR (500 MHz, DMSO-d_6): δ 11.88 (s, 1H, 3-NH), 8.17 (d, *J*=6.8 Hz, 1H, 6-H), 6.77 (qd, *J*=6.3, 1.5 Hz, 1H, N-C$\underline{H}$(CH_3)-O), 5.18 (d, *J*=3.8 Hz, 2H, C1′-H, C4′-OH of sucrose), 5.14 (d, *J*=6.3 Hz, 1H, C3′-OH of sucrose), 5.02 (s, 1H, C2′-OH of sucrose), 4.91 (d, *J*=8.6 Hz, 1H, C3′′-OH of sucrose), 4.82 (t, *J*=6.4 Hz, 1H, C1′′-OH of sucrose), 4.58 (d, *J*=7.9 Hz, 1H, C4′′-OH of sucrose), 4.41 (t, *J*=5.5 Hz, 1H, C6′′-OH of sucrose), 4.24 (dt, *J*=11.7, 2.2 Hz, 1H, C6′-Ha of sucrose), 4.02 (ddd, *J*=11.7, 6.1, 2.7 Hz, 1H, C5′-H of sucrose), 3.94-3.85 (m, 2H, C3′′-H, C6′-Hb of sucrose), 3.73 (dt, *J*=8.0, 4.3 Hz, 1H, C5′′-H of sucrose), 3.62-3.43 (m, 4H, C4′′-H, C3′-H, C6′′-Ha, C6′′-Hb of sucrose), 3.34-2.97 (m, 4H, C1′′-Ha, C1′′-Hb, C2′-H, C4′-H of sucrose), 2.34 (m, 4H, -C$\underline{H_2}$-C$\underline{H_2}$-), 1.53 (dd, *J*=8.4, 4.8 Hz, 7H, -CH(C$\underline{H_3}$)-O-C=O-C$\underline{H_2}$-, -C$\underline{H_2}$-C=O-O-). ^{13}C NMR (126 MHz, DMSO-d_6): δ 172.80 (C-8), 171.16 (C-13), 157.14, 156.93 (C-4), 148.47 (C-2), 141.22, 139.38(C-5), 125.01, 124.74(C-6), 103.92 (C-2′′ of sucrose), 91.50 (C-1′ of sucrose), 82.73 (C-5′′ of sucrose), 77.01 (C-3′′ of sucrose), 75.19 (C-7), 74.56 (C-4′′ of sucrose), 72.72 (C-3′ of sucrose), 71.55 (C-2′ of sucrose), 70.21 (C-5′ of sucrose), 70.03 (C-4′ of sucrose), 63.71 (C-6′ of sucrose), 62.63(C-1′′ of sucrose), 62.25 (C-6′′ of sucrose), 32.94, 32.88 (C-9, C-12), 23.69, 23.60 (C-10, C-11), 18.47 (C-14). HRMS: $C_{24}H_{35}FN_2NaO_{16}$ for $[M+Na]^+$, calculated 649.1940, found 649.1349.

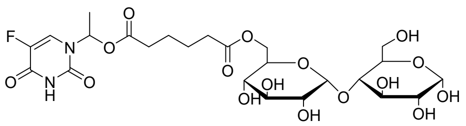

6′′-O-[1-(1-(5-Fluououracil))-ethyl vinyl adipate]-maltose (5d): white solid, isolated yield 36.1%; ^{1}H NMR (500 MHz, DMSO-d_6): δ 11.88 (s, 1H, 3-NH), 8.17 (d, *J*=6.8 Hz, 1H, 6-H), 6.77 (qd, *J*=6.2, 1.5 Hz, 1H, N-C<u>H</u>(CH_3)-O), 6.68 (d, *J*=6.2 Hz, 0.5H, C1′-OH of β-maltose), 6.35 (d, *J*=4.4 Hz, 0.5H, C1′-OH of α-maltose), 5.54 (t, *J*=6.6 Hz, 1H, C2′′-OH of β-maltose, C2′′-OH of α-maltose), 5.49 (d, *J*=2.8 Hz, 0.5H, C3′-OH of β-maltose), 5.34 (d, *J*=3.0 Hz, 0.5H, C3′-OH of α-maltose), 5.17 (s, 1H, C4′′-OH of β-maltose, C4′′-OH of α-maltose), 4.98 (dd, *J*=13.5, 3.8 Hz, 2.5H, C3′′-OH, C1′′-H of β-maltose, C3′′-OH, C1′′-H, C1′-H of α-maltose), 4.92 (d, *J*=3.3 Hz, 0.5H, C2′-OH of β-maltose), 4.63 (d, *J*=6.8 Hz, 0.5H, C2′-OH of α-maltose), 4.49 (t, *J*=6.0 Hz, 0.5H, C6′-OH of β-maltose), 4.37 (d, *J*=6.1 Hz, 0.5H, C6′-OH of α-maltose), 4.34-4.24 (m, 1.5H, C6′′-Ha, C1′-H of β-maltose, C6′′-Ha of α-maltose), 4.01 (ddd, *J*=11.3, 6.7, 2.0 Hz, 1H, C6′′-Hb of β-maltose, C6′′-Hb of α-maltose), 3.74-3.60 (m, 3H, C6′-Ha, C6′-Hb, C5′′-H of β-maltose, C6′-Ha, C6′-Hb, C5′′-H of α-maltose), 3.53 (m, 1H, C5′-H of β-maltose, C5′-H of α-maltose), 3.36 (s, 0.5H, C3′′-H of β-maltose), 3.30-3.12 (m, 4H, C3′-H, C4′-H, C2′′-H of β-maltose, C3′′-H, C3′-H, C4′-H, C2′′-H, C2′-H of α-maltose), 3.05 (t, *J*=9.5 Hz, 1H, C4′′-H of β-maltose, C4′′-H of α-maltose), 2.96 (t, *J*=8.8 Hz, 0.5H, C2′-H of β-maltose), 2.34 (m, 4H, -C<u>H</u>$_2$-C<u>H</u>$_2$-), 1.53 (dd, *J*=9.4, 5.0 Hz, 7H, -CH(C<u>H</u>$_3$)-O-C=O-C<u>H</u>$_2$-, -C<u>H</u>$_2$-C=O-O-). ^{13}C NMR (126 MHz, $CDCl_3$): δ 172.75 (C-8), 171.19 (C-13), 157.17, 156.96 (C-4), 148.49 (C-2), 141.24, 139.40 (C-5), 125.05, 124.78 (C-6), 101.10, 101.01 (C-1′′ of β-maltose, C-1′′ of α-maltose), 96.81 (C-1′ of β-maltose), 92.13 (C-1′ of α-maltose), 81.15 (C-4′ of α-maltose), 80.60 (C-4′ of β-maltose), 76.55 (C-3′ of β-maltose), 75.21 (C-7, C-5′ of β-maltose), 74.31 (C-2′ of β-maltose), 73.24, 73.20 (C-3′ of α-maltose, C-3′′ of α-maltose), 72.97 (C-3′′ of β-maltose), 72.55 (C-2′′ of β-maltose), 72.42 (C-2′′ of α-maltose), 71.85 (C-5′′ of α-maltose), 70.66 (C-5′′ of β-maltose), 70.62 (C-2′ of α-maltose), 70.44 (C-5′ of α-maltose), 70.11 (C-4′′ of α-maltose), 70.07 (C-4′′ of β-maltose), 63.68 (C-6′′ of α-maltose, C-6′′ of β-maltose), 60.82 (C-6′ of β-maltose), 60.70 (C-6′ of α-maltose), 32.95, 32.89 (C-9, C-12), 23.68, 23.62 (C-10, C-11), 18.50 (C-14). HRMS: $C_{24}H_{35}FN_2NaO_{16}$ for $[M+Na]^+$, calculated 649.1940, found 649.1347.

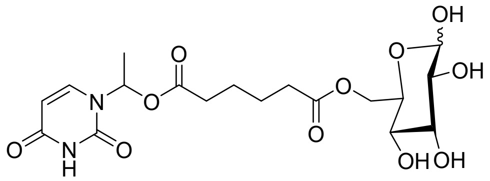

6′-O-[1-(1-uracil))-ethyl vinyl adipate]- D-glucose (5e): white solid, isolated yield 52.7%; ^{1}H NMR (500 MHz, DMSO-d_6): δ 11.37 (s, 1H, 3- NH), 7.77 (dd, *J*=8.0, 1.7 Hz, 1H, 6-H), 6.78 (q, *J*=6.2 Hz, 1H, N-CH(CH_3)-O), 6.67 (dd, *J*=6.6, 1.1 Hz, 0.5H, C1′-OH of β-D-glucose), 6.35 (dd, *J*=4.8, 0.9 Hz, 0.5H, C1′-OH of α-D-glucose), 5.66 (d, *J*=8.0 Hz, 1H, C-5), 5.10 (d, *J*=5.4 Hz, 0.5H, C1′-H of β-D-glucose), 5.05 (d, *J*=5.7 Hz, 0.5H, C1′-H of α-D-glucose), 4.95 (d, *J*=4.8 Hz, 0.5 H, C4′-OH of β-D-glucose), 4.90 (dd, *J*=7.8, 4.5 Hz, 1H, C2′-OH of β-D-glucose, C4′-OH of α-D-glucose), 4.75 (d, *J*=4.8 Hz, 0.5H, C2′-OH of α-D-glucose), 4.53 (d, *J*=6.7 Hz, 0.5H, C3′-OH of α-D-glucose), 4.34-4.24 (m, 1.5H, C6′-Ha, C3′-OH of β-D-glucose, C6′-Ha of α-D-glucose), 4.04-3.94 (m, 1H, C6′-Hb of β-D-glucose, C6′-Hb of α-D-glucose), 3.77 (ddd, *J*=10.0, 6.3, 2.0 Hz, 0.5H, C5′-H of α-D-glucose), 3.43 (td, *J*=9.2, 4.5 Hz, 0.5H, C5′-H of β-D-glucose), 3.32-3.30 (m, 1H, 0.5H, C3′-H of α-D-glucose), 3.13 (td, *J*=8.7, 4.4

Hz, 1H, C2′-H of β-D-glucose, C2′-H of α-D-glucose), 3.04 (ddt, *J*=15.5, 11.8, 5.7 Hz, 1H, C3′-H of β-D-glucose, C4′-H of α-D-glucose), 2.90 (ddd, *J*=9.0, 7.7, 4.5 Hz, 0.5H, C4′-H of β-D-glucose), 2.38 – 2.27 (m, 4H, -C$\underline{H}_2$-C$\underline{H}_2$-), 1.58-1.48 (m, 7H, -CH(C$\underline{H}_3$)-O-C=O-C$\underline{H}_2$-, -C$\underline{H}_2$-C=O-O-). ^{13}C NMR (126 MHz, $CDCl_3$): δ 172.67 (C-8), 171.11 (C-13), 162.92 (C-4), 149.72 (C-2), 140.37 (C-6), 102.33 (C-5), 96.86 (C-1′ of β-D-glucose), 92.26 (C-1′ of α-D-glucose), 76.39 (C-3′ of β-D-glucose), 74.92 (C-7), 74.68 (C-2′ of β-D-glucose), 73.47 (C-5′ of β-D-glucose), 72.85 (C-3′ of α-D-glucose), 72.16 (C-2′ of α-D-glucose), 70.56 (C-4′ of α-D-glucose), 70.15 (C-4′ of β-D-glucose), 69.11 (C-5′ of α-D-glucose), 63.93 (C-6′), 32.98, 32.93, 32.83 (9-C, 12-C), 23.66, 23.52 (10-C, 11-C), 18.60 (14-C). HRMS: $C_{18}H_{26}N_2NaO_{11}$ for $[M+Na]^+$, calculated 469.1432, found 469.1443.

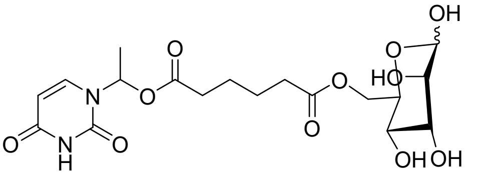

6′-O-[1-(1-uracil))-ethyl vinyl adipate]- D-mannose (5f): white solid, isolated yield 55.4%; ^{1}H NMR (500 MHz, DMSO-*d*$_6$): δ 11.36 (s, 1H, 3-NH), 7.76 (d, *J*=8.0 Hz, 1H, 6-H), 6.78 (q, *J*=6.2 Hz, 1H, N-CH(CH_3)-O), 6.36 (t, *J*=3.2 Hz, 1H, C1′-OH of D-Mannose), 5.66 (d, *J*=8.0 Hz, 1H, 5-H), 4.90-4.84 (m, 2H, C1′-H, C4′-OH of D-Mannose), 4.62 (d, *J*=3.8 Hz, 1H, C3′-OH of D-Mannose), 4.56 (m, 1H, C2′-OH of D-Mannose), 4.30 (dd, *J*=11.6, 2.0 Hz, 1H, C6′-H of D-Mannose), 4.01 (dd, *J*=11.6, 6.8 Hz, 1H, C6′-H of D-Mannose), 3.71 (ddd, *J*=9.1, 6.8, 1.9 Hz, 1H, C5′-H of D-Mannose), 3.57-3.50 (m, 2H, C2′-H, C3′-H of D-Mannose), 3.38 (s, 1H, C4′-H of D-Mannose), 2.32 (m, 4H, -C$\underline{H}_2$-C$\underline{H}_2$-), 1.53 (dd, *J*=11.2, 4.9 Hz, 7H, -CH(C$\underline{H}_3$)-O-C=O-C$\underline{H}_2$-, -C$\underline{H}_2$-C=O-O-). ^{13}C NMR (126 MHz, DMSO-*d*$_6$): δ 172.78 (C-8), 171.20 (C-13), 163.00 (C-4), 149.78 (C-2), 140.42 (C-6), 102.38 (C-5), 94.08 (C-1′ of D-Mannose), 74.97 (C-2′ of D-Mannose), 71.32 (C-3′ of D-Mannose), 70.40(C-5′ of D-Mannose), 67.16 (C-4′ of D-Mannose), 64.24 (C-6′ of D-Mannose), 33.07, 32.89 (9-C, 12-C), 23.73, 23.59 (10-C, 11-C), 18.65 (14-C). HRMS: $C_{18}H_{26}N_2NaO_{11}$ for $[M+Na]^+$, calculated 469.1432, found 469.1439.

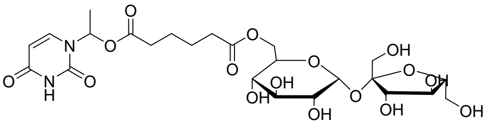

6′-O-[1-(1-uracil))-ethyl vinyl adipate]- sucrose (5g): white solid, isolated yield 37.2%; ^{1}H NMR (500 MHz, DMSO-*d*$_6$): δ 11.36 (s, 1H, 3-NH), 7.76 (d, *J*=8.1 Hz, 1H, 6-H), 6.78 (q, *J*=6.2 Hz, 1H, N-CH(CH_3)-O), 5.66 (d, *J*=8.0 Hz, 1H, C-5), 5.22-5.15 (m, 2H, C1′-H, C4′-OH of sucrose), 5.13 (d, *J*=6.0 Hz, 1H, C3′-OH of sucrose), 5.02 (d, *J*=5.5 Hz, 1H, C2′-OH of sucrose), 4.90 (s, 1H, C3′′-OH of sucrose), 4.81 (t, *J*=6.4 Hz, 1H, C1′′-OH of sucrose), 4.58 (d, *J*=7.9 Hz, 1H, C4′′-OH of sucrose), 4.41 (t, *J*=5.3 Hz, 1H, C6′′-OH of sucrose), 4.24 (dt, *J*=11.8, 2.0 Hz, 1H, C6′-Ha of sucrose), 4.02 (ddd, *J*=11.7, 6.1, 2.3 Hz, 1H, C5′-H of sucrose), 3.94-3.85 (m, 2H, C3′′-H, C6′-Hb of sucrose), 3.73 (td, *J*=7.7, 5.5 Hz, 1H, C5′′-H of sucrose), 3.63-3.44 (m, 4H, C4′′-H, C3′-H, C6′′-Ha, C6′′-Hb of sucrose), 3.39 (m, 2H, C1′′-Ha, C1′′-Hb of sucrose), 3.24-3.03 (m, 2H, C2′-H, C4′-H of sucrose), 2.34 (m, 4 H, -C$\underline{H}_2$-C$\underline{H}_2$-), 1.53 (dd, *J*=11.7, 4.9 Hz, 7H, -CH(C$\underline{H}_3$)-O-C=O-C$\underline{H}_2$-, -C$\underline{H}_2$-C=O-O-). ^{13}C NMR (126 MHz, DMSO-*d*$_6$): δ 172.75 (C-8), 171.15 (C-13), 162.97 (C-4), 149.76 (C-2), 140.37 (C-6), 103.90 (C-2'' of sucrose), 102.37 (5-C), 91.47 (C-1′ of sucrose), 82.72 (C-5′′ of sucrose), 76.95 (C-3′′ of sucrose), 74.94 (C-7), 74.52 (C-4′′ of sucrose), 72.68 (C-3′ of sucrose), 71.52 (C-2′ of sucrose), 70.17 (C-5′ of

sucrose), 69.99 (C-4′ of sucrose), 63.67 (C-6′ of sucrose), 62.60 (C-1′′ of sucrose), 62.20 (C-6′′ of sucrose), 32.90, 32.87 (C-9, C-12), 23.65, 23.61 (C-10, C-11), 18.63 (C-14). HRMS: $C_{24}H_{36}N_2NaO_{16}$ for $[M+Na]^+$, calculated 631.2039, found 631.2050.

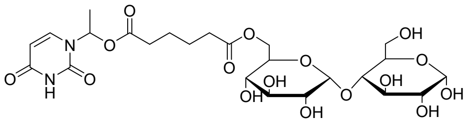

6′-O-[1-(1-uracil))-ethyl vinyl adipate] -maltose (5h): white solid, isolated yield 36.3%; ^{1}H NMR (500 MHz, DMSO-d_6): δ 11.35 (s, 1H, 3-NH), 7.75 (d, *J*=8.0 Hz, 1H, 6-H), 6.77 (q, *J*=6.2 Hz, 1H, N-CH(CH_3)-O), 6.68 (d, *J*=6.5 Hz, 0.5H, C1′-OH of β-maltose), 6.35 (d, *J*=4.6 Hz, 0.5H, C1′-OH of α-maltose), 5.66 (d, *J*=8.0 Hz, 1H, C-5), 5.54 (t, *J*=6.8 Hz, 1H, C2′′-OH of -maltose, C2′′-OH of α-maltose), 5.49 (d, *J*=2.8 Hz, 0.5H, C3′-OH of β-maltose), 5.34 (d, *J*=3.0 Hz, 0.5H, C3′-OH of α-maltose), 5.17 (t, *J*=6.1 Hz, 1H, C4′′-OH of β-maltose, C4′′-OH of α-maltose), 5.06-4.94 (m, 2.5H, C3′′-OH, C1′′-H of β-maltose, C3′′-OH, C1′′-H, C1′-H of α-maltose), 4.92 (t, *J*=4.0 Hz, 0.5H, C2′-OH of β-maltose), 4.63 (d, *J*=6.8 Hz, 0.5H, C2′-OH of α-maltose), 4.50 (t, *J*=6.0 Hz, 0.5H, C6′-OH of β-maltose), 4.39 (t, *J*=5.9 Hz, 0.5H, C6′-OH of α-maltose), 4.35-4.22 (m, 1.5H, C6′′-Ha, C1′-H of β-maltose, C6′′-Ha of α-maltose), 4.01 (ddd, *J*=11.6, 6.7, 2.4 Hz, 1H, C6′′-Hb of β-maltose, C6′′-Hb of α-maltose), 3.76-3.61 (m, 3H, C6′-Ha, C6′-Hb, C5′′-H of β-maltose, C6′-Ha, C6′-Hb, C5′′-H of α-maltose), 3.53 (ddd, *J*=26.0, 12.0, 5.8 Hz, 1H, C5′-H of β-maltose, C5′-H of α-maltose), 3.37-3.35 (m, 1.5H, C3′′-H of β-maltose, C3′′-H, C3′-H of α-maltose), 3.29-3.16 (m, 3H, C3′-H, C4′-H, C2′′-H of β-maltose, C4′-H, C2′′-H, C2′-H of α-maltose), 3.06 (td, *J*=9.4, 4.9 Hz, 1H, C4′′-H of β-maltose, C4′′-H of α-maltose), 2.96 (td, *J*=8.5, 4.1 Hz, 0.5H, C2′-H of β-maltose), 2.40-2.27 (m, 4H, -C$\underline{H_2}$-C$\underline{H_2}$-), 1.53 (dd, *J*=13.1, 5.3 Hz, 7H, -CH(C$\underline{H_3}$)-O-C=O-C$\underline{H_2}$-, -C$\underline{H_2}$-C=O-O-). ^{13}C NMR (126 MHz, $CDCl_3$): δ 172.75 (C-8), 171.23 (C-13), 163.06 (C-4), 149.82 (C-2), 140.44 (C-6), 102.44(5-C), 101.10, 101.01 (C-1′′ of β-maltose, C-1′′ of α-maltose), 96.81 (C-1′ of β-maltose), 92.13 (C-1′ of α-maltose), 81.14 (C-4′ of α-maltose), 80.60 (C-4′ of β-maltose), 76.56 (C-3′ of β-maltose), 75.22 (C-5′ of β-maltose), 75.00 (C-7), 74.32 (C-2′ of β-maltose), 73.25, 73.21 (C-3′ of α-maltose, C-3′′ of α-maltose), 72.98 (C-3′′ of β-maltose), 72.55 (C-2′′ of β-maltose), 72.42 (C-2′′ of α-maltose), 71.86 (C-5′′ of α-maltose), 70.67, 70.63 (C-5′′ of β-maltose, C-2′ of α-maltose), 70.44 (C-5′ of α-maltose), 70.12, 70.08 (C-4′′ of α-maltose, C-4′′ of β-maltose), 63.70 (C-6′′ of α-maltose, C-6′′ of β-maltose), 60.84 (C-6′ of β-maltose), 60.71 (C-6′ of α-maltose), 32.95, 32.92 (C-9, C-12), 23.69, 23.67 (C-10, C-11), 18.70 (C-14). HRMS: $C_{24}H_{36}N_2NaO_{16}$ for $[M+Na]^+$, calculated 631.2039, found 631.2045.

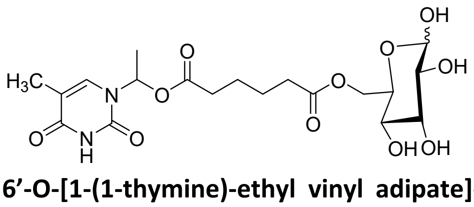

6′-O-[1-(1-thymine)-ethyl vinyl adipate]- D-glucose (5i): white solid, isolated yield 51.8%; ^{1}H NMR (500 MHz, DMSO-d_6): δ 11.34 (s, 1H, 3-NH), 7.64 (q, *J*=1.3 Hz, 1H, 6-H), 6.80 (q, *J*=6.2 Hz, 1H, N-CH(CH_3)-O), 6.66 (s, 0.5H, C1′-OH of β-D-glucose), 6.34 (s, 0.5H, C1′-OH of α-D-glucose), 5.10 (s, 0.5H, C1′-H of β-D-glucose), 5.04 (s, 0.5H, C1′-H of α-D-glucose), 4.98-4.87 (m, 1.5H, C4′-OH, C2′-OH of β-D-glucose, C4′-OH of α-D-glucose), 4.75 (s, 0.5H, C2′-OH of α-D-glucose), 4.53 (s, 0.5H, C3′-OH of α-D-glucose), 4.30 (ddd, *J*=11.4, 4.9, 3.3 Hz, 1.5H, C6′-Ha, C3′-OH of β-D-glucose, C6′-Ha of α-D-glucose), 4.03-3.95 (m, 1H, C6′-Hb of β-D-glucose, C6′-Hb of α-D-glucose), 3.77 (ddd,

J=10.0, 6.2, 2.0 Hz, 0.5H, C5′-H of α-D-glucose), 3.43 (t, *J*=9.1 Hz, 0.5H, C5′-H of β-D-glucose), 3.31 (dd, *J*=6.8, 2.1 Hz, 0.5H, C3′-H of α-D-glucose), 3.14 (d, *J*=9.0 Hz, 1H, C2′-H of β-D-glucose, C2′-H of α-D-glucose), 3.05 (dd, *J*=12.1, 5.3 Hz, 1H, C3′-H of β-D-glucose, C4′-H of α-D-glucose), 2.91 (t, *J*=8.4 Hz, 0.5H, C4′-H of β-D-glucose), 2.38-2.27 (m, 4H, -C$\underline{H}_2$-C$\underline{H}_2$-), 1.80 (d, *J*=1.2 Hz, 3H, 5-C$\underline{H}_3$), 1.53 (dd, *J*=10.5, 5.9 Hz, 7H, -CH(C$\underline{H}_3$)-O-C=O-C$\underline{H}_2$-, -C$\underline{H}_2$-C=O-O-). ^{13}C NMR (126 MHz, $CDCl_3$): δ 172.68 (C-8), 171.12 (C-13), 163.62 (C-4), 149.76 (C-2), 135.65 (C-2), 110.13 (C-6), 96.87 (C-1′ of β-D-glucose), 92.27 (C-1′ of α-D-glucose), 76.40 (C-3′ of β-D-glucose), 74.69 (C-2′ of β-D-glucose, C-7), 73.48 (C-5′ of β-D-glucose), 72.86 (C-3′ of α-D-glucose), 72.17 (C-2′ of α-D-glucose), 70.57 (C-4′ of α-D-glucose), 70.16 (C-4′ of β-D-glucose), 69.11 (C-5′ of α-D-glucose), 63.95 (C-6′), 33.00, 32.95, 32.88 (C-9, C-12), 23.69, 23.57 (C-10, C-11), 18.62 (14-C), 12.00 (15-C). HRMS: $C_{19}H_{28}N_2NaO_{11}$ for $[M+Na]^+$, calculated 483.1625, found 483.1636.

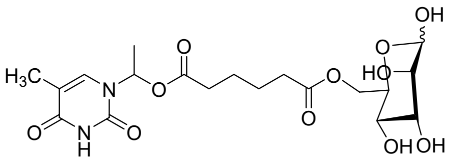

6′-O-[1-(1-thymine)-ethyl vinyl adipate]- D-mannose (5j): white solid, isolated yield 50.3%; ^{1}H NMR (500 MHz, DMSO-d_6): δ 11.33 (s, 1H, 3-NH), 7.70-7.58 (m, 1H, 6-H), 6.80 (q, *J*=6.2 Hz, 1H, N-CH(CH_3)-O), 6.34 (d, *J*=5.0 Hz, 1H, C1′-OH of D-Mannose), 4.86 (d, *J*=3.9 Hz, 2H, C1′-H, C4′-OH of D-Mannose), 4.59 (s, 1H, C3′-OH of D-Mannose), 4.52 (s, 1H, C2′-OH of D-Mannose), 4.30 (dd, *J*=11.7, 2.0 Hz, 1H, C6′-H of D-Mannose), 4.04-3.98 (m, 1H, C6′-H of D-Mannose), 3.71 (td, *J*=7.1, 3.4 Hz, 1H, C5′-H of D-Mannose), 3.59-3.50 (m, 2H, C2′-H, C3′-H of D-Mannose), 3.38 (td, *J*=9.5, 4.7 Hz, 1H, C4′-H of D-Mannose), 2.38-2.27 (m, 4H, -C$\underline{H}_2$-C$\underline{H}_2$-), 1.80 (d, *J*=1.2 Hz, 3H, 5-C$\underline{H}_3$), 1.54 (dd, *J*=10.0, 5.2 Hz, 7H, -CH(C$\underline{H}_3$)-O-C=O-C$\underline{H}_2$-, -C$\underline{H}_2$-C=O-O-). ^{13}C NMR (126 MHz, DMSO-d_6): δ 172.71 (C-8), 171.14 (C-13), 163.61 (C-4), 149.75 (C-2), 135.64 (C-6), 110.12 (C-5), 94.04 (C-1′ of D-Mannose), 74.69 (C-2′ of D-Mannose), 71.28 (C-3′ of D-Mannose), 70.36 (C-5′ of D-Mannose), 67.14 (C-4′ of D-Mannose), 64.21 (C-6′ of D-Mannose), 33.04, 32.89 (9-C, 12-C), 23.69, 23.58 (10-C, 11-C), 18.61 (14-C), 12.00 (15-C). HRMS: $C_{19}H_{28}N_2NaO_{11}$ for $[M+Na]^+$, calculated 483.1625, found 483.1631.

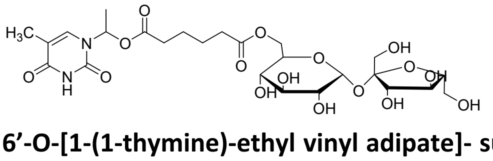

6′-O-[1-(1-thymine)-ethyl vinyl adipate]- sucrose (5k): white solid, isolated yield 35.9%; ^{1}H NMR (500 MHz, DMSO-d_6): δ 11.32 (s, 1H, 3-NH), 7.67-7.60 (m, 1H, 6-H), 6.79 (q, *J*=6.2 Hz, 1H, N-CH(CH_3)-O), 5.18 (d, *J*=3.7 Hz, 1H, C1′-H, C4′-OH of sucrose), 5.13 (d, *J*=3.9 Hz, 1H, C3′-OH of sucrose), 5.01 (s, 1H, C2′-OH of sucrose), 4.90 (s, 1H, C3′′-OH of sucrose), 4.81 (s, 1H, 1H, C1′′-OH of sucrose), 4.56 (s, 1H, C4′′-OH of sucrose), 4.40 (s, 1H, C6′′-OH of sucrose), 4.02 (ddd, *J*=11.7, 6.1, 2.5 Hz, 1H, C5′-H of sucrose), 3.95-3.85 (m, 2H, C3′′-H, C6′-Hb of sucrose), 3.73 (d, *J*=8.8 Hz, 1H, C5′′-H of sucrose), 3.62-3.45 (m, 4H, C4′′-H, C3′-H, C6′′-Ha, C6′′-Hb of sucrose), 3.34-2.86 (m, 4H, C1′′-Ha, C1′′-Hb, C2′-H, C4′-H of sucrose), 2.38-2.29 (m, 4H, -C$\underline{H}_2$-C$\underline{H}_2$-), 1.80 (d, *J*=1.2 Hz, 3H, 5-C$\underline{H}_3$), 1.53 (dd, *J*=10.5, 5.1 Hz, 7H, -CH(C$\underline{H}_3$)-O-C=O-C$\underline{H}_2$-, -C$\underline{H}_2$-C=O-O-). ^{13}C NMR (126 MHz, DMSO-d_6): δ 172.77 (C-8), 171.18 (C-13), 163.67 (C-4), 149.80 (C-2), 135.68 (C-6), 110.19 (5-C), 103.91 (C-2′′ of sucrose), 91.49 (C-1′ of sucrose), 82.72 (C-5′′ of sucrose), 77.00 (C-3′′ of sucrose),

74.74 (C-7), 74.55 (C-4'' of sucrose), 72.71 (C-3' of sucrose), 71.54 (C-2' of sucrose), 70.19 (C-5' of sucrose), 70.01 (C-4' of sucrose), 63.69 (C-6' of sucrose), 62.62 (C-1'' of sucrose), 62.24 (C-6'' of sucrose), 32.93 (C-9, C-12), 23.67 (C-10, C-11), 18.66 (C-14), 12.04 (C-15). HRMS: $C_{25}H_{38}N_2NaO_{16}$ for $[M+Na]^+$, calculated 645.2130, found 645.2146.

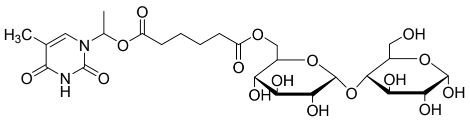

6'-O-[1-(1-thymine)-ethyl vinyl adipate]- maltose (5l): white solid, isolated yield 38.3%; ^1H NMR (500 MHz, DMSO-d_6): δ 11.32 (s, 1H, 3-NH), 7.62 (d, *J*=2.8 Hz, 1H, 6-H), 6.79 (q, *J*=6.2 Hz, 1H, N-CH(CH_3)-O), 6.66 (d, *J*=6.4 Hz, 0.5H, C1'-OH of β-maltose), 6.34 (d, *J*=4.5 Hz, 0.5H, C1'-OH of α-maltose), 5.52 (s, 1H, C2''-OH of β-maltose, C2''-OH of α-maltose), 5.49-5.44 (m, 0.5H, C3'-OH of β-maltose), 5.33 (s, 0.5H, C3'-OH of α-maltose), 5.16 (s, 1H, C4''-OH of β-maltose, C4''-OH of α-maltose), 4.98 (dd, *J*=13.0, 3.8 Hz, 2.5H, C3''-OH, C1''-H of β-maltose, C3''-OH, C1''-H, C1'-H of α-maltose), 4.92 (d, *J*=3.7 Hz, 0.5H, C2'-OH of β-maltose), 4.61 (s, 0.5H, C2'-OH of α-maltose), 4.48 (d, *J*=6.5 Hz, 0.5H, C6'-OH of β-maltose), 4.40-4.34 (m, 0.5H, C6'-OH of α-maltose), 4.34-4.24 (m, 1.5H, C6''-Ha, C1'-H of β-maltose, C6''-Ha of α-maltose), 4.02 (dd, *J*=11.8, 6.8 Hz, 1H, C6''-Hb of β-maltose, C6''-Hb of α-maltose), 3.78-3.60 (m, 3H, C6'-Ha, C6'-Hb, C5''-H of β-maltose, C6'-Ha, C6'-Hb, C5''-H of α-maltose), 3.53 (m, 1H, C5'-H of β-maltose, C5'-H of α-maltose), 3.37 (s, 1.5H, C3''-H of β-maltose, C3''-H, C3'-H of α-maltose), 3.30-3.15 (m, 3H, C3'-H, C4'-H, C2''-H of β-maltose, C4'-H, C2''-H, C2'-H of α-maltose), 3.06 (t, *J*=9.2 Hz, 1H, C4''-H of β-maltose, C4''-H of α-maltose), 3.01-2.91 (m, 0.5H, C2'-H of β-maltose), 2.33 (m, 4H, -C$\underline{H_2}$-C$\underline{H_2}$-), 1.80 (t, *J*=1.1 Hz, 3H, 5-C$\underline{H_3}$), 1.60-1.47 (m, 7H, -CH(C$\underline{H_3}$)-O-C=O-C$\underline{H_2}$-, -C$\underline{H_2}$-C=O-O-). ^{13}C NMR (126 MHz, DMSO-d_6): δ 172.70 (C-8), 171.18 (C-13), 163.68 (C-4), 149.81 (C-2), 135.68 (C-6), 110.20 (5-C), 101.07, 100.98 (C-1'' of β-maltose, C-1'' of α-maltose), 96.79 (C-1' of β-maltose), 92.10 (C-1' of α-maltose), 81.11 (C-4' of α-maltose), 80.58 (C-4' of β-maltose), 76.53 (C-3' of β-maltose), 75.20 (C-5' of β-maltose), 74.76 (C-7), 74.30 (C-2' of β-maltose), 73.23, 73.18 (C-3' of α-maltose, C-3'' of α-maltose), 72.95 (C-3'' of β-maltose), 72.53 (C-2'' of β-maltose), 72.40 (C-2'' of α-maltose), 71.84 (C-5'' of α-maltose), 70.64, 70.60 (C-5'' of β-maltose, C-2' of α-maltose), 70.42 (C-5' of α-maltose), 70.10, 70.06 (C-4'' of α-maltose, C-4'' of β-maltose), 63.67, 63.64 (C-6'' of α-maltose, C-6'' of β-maltose), 60.81 (C-6' of β-maltose), 60.69 (C-6' of α-maltose), 32.94 (C-9, C-12), 23.67 (C-10, C-11), 18.67 (C-14), 12.05 (C-15). HRMS: $C_{25}H_{38}N_2NaO_{16}$ for $[M+Na]^+$, calculated 645.2130, found 645.2141.

6'-O-[1-(1-(5-Fluorouracil))-ethyl vinyl adipate]-D-glucose (5a)

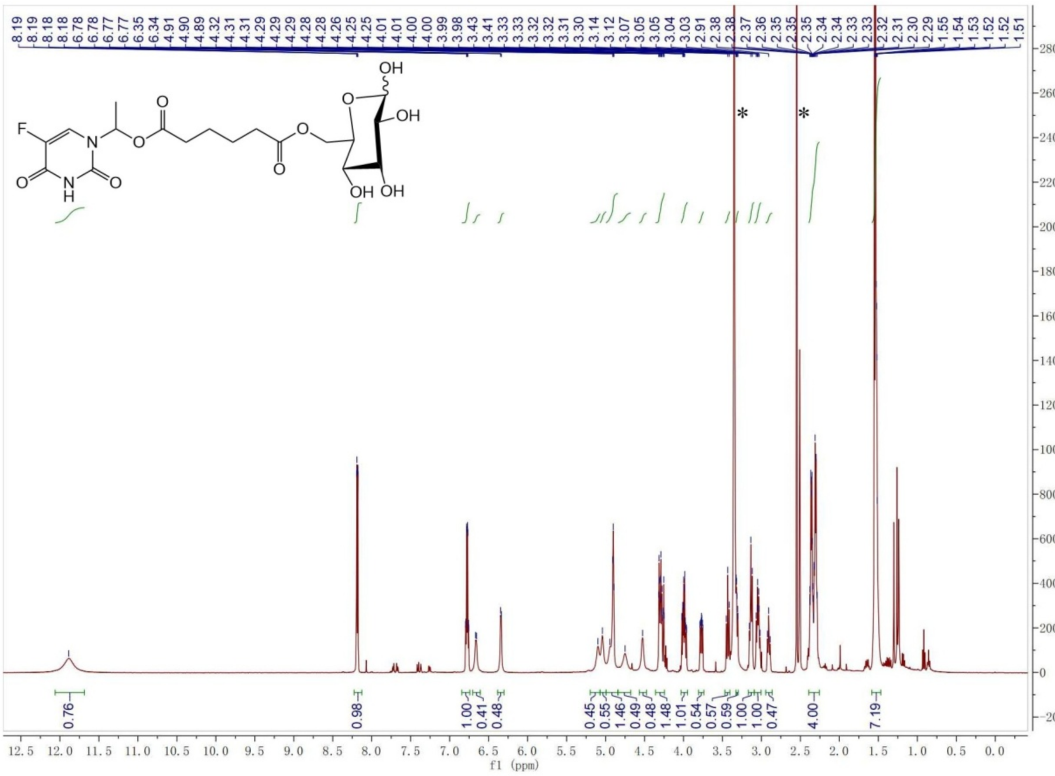

6’-O-[1-(1-(5-Fluououracil))-ethyl vinyl adipate]-D-mannose (5b)

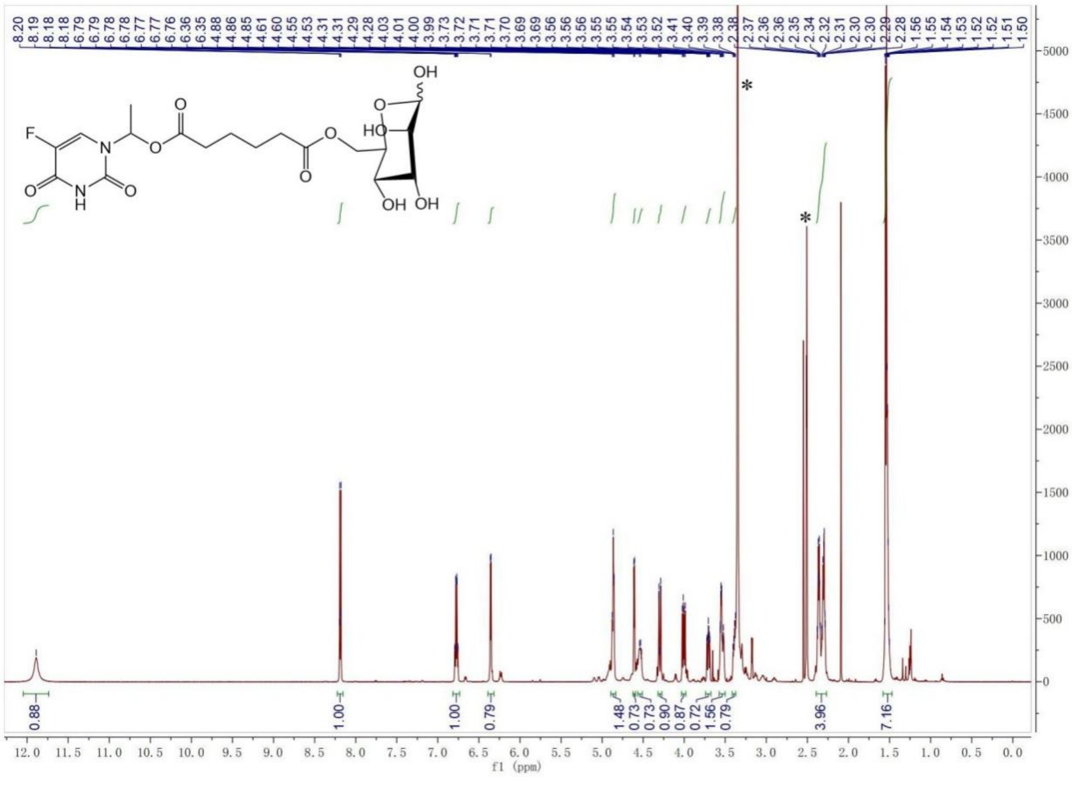

6’-O-[1-(1-(5-Fluououracil))-ethyl vinyl adipate]-sucrose (5c)

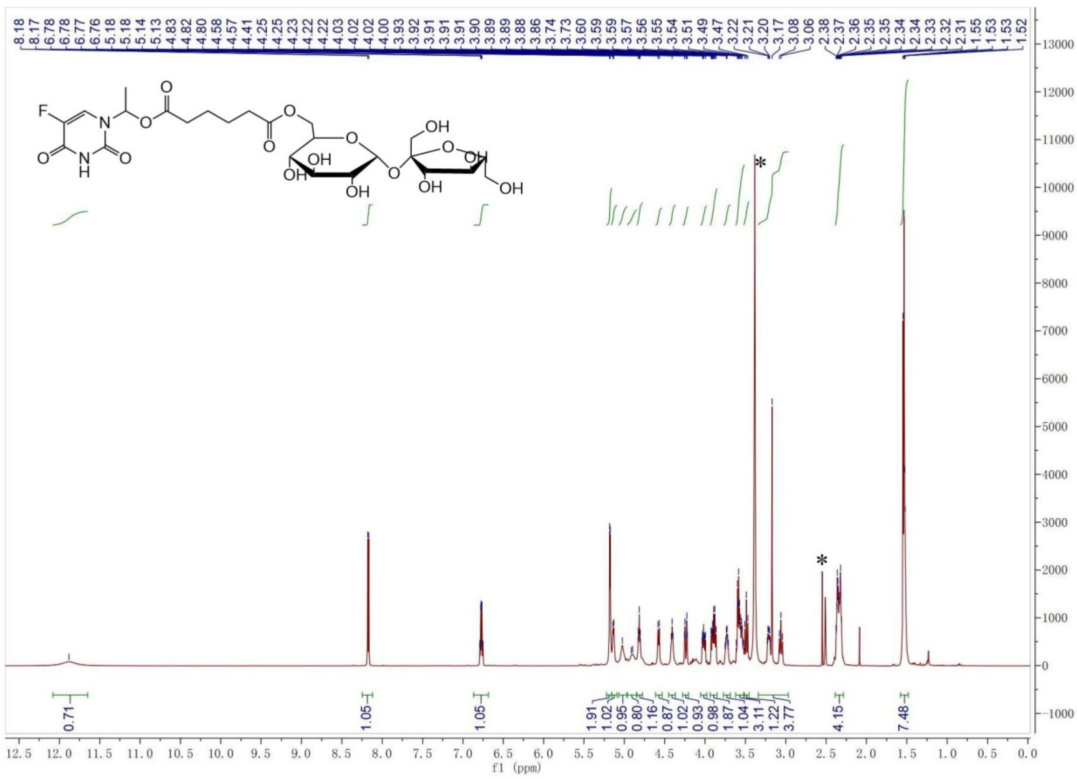

6’-O-[1-(1-(5-Fluououracil))-ethyl vinyl adipate]-maltose (5d)

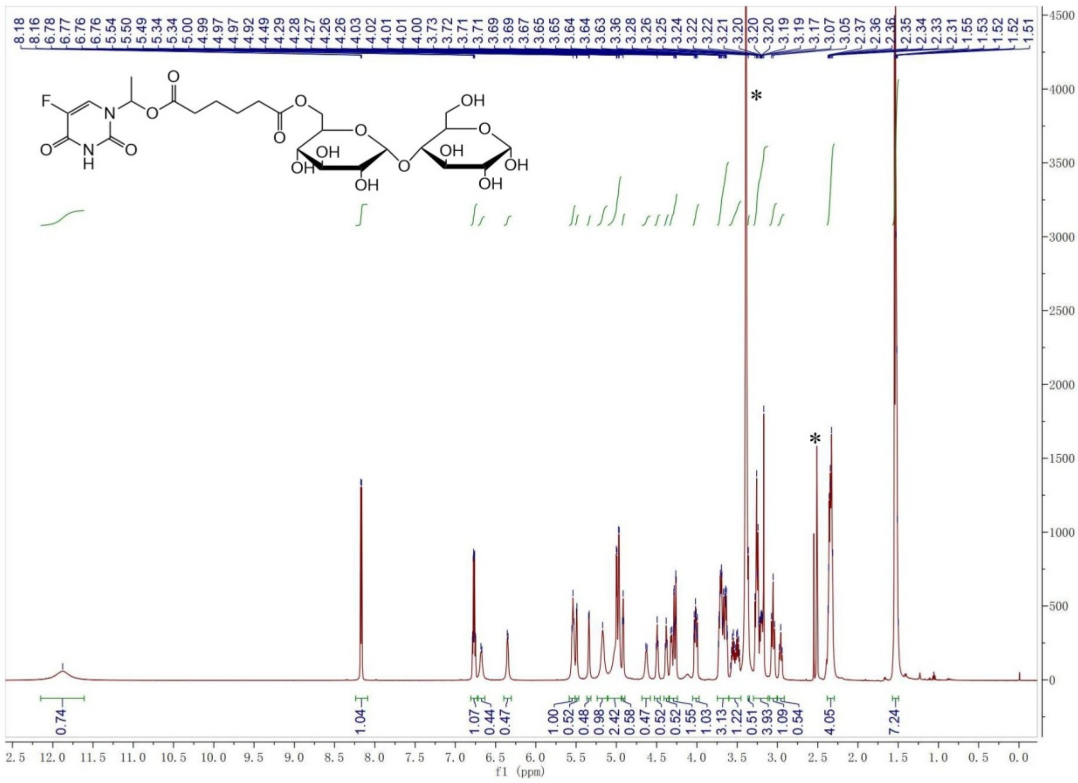

6’-O-[1-(1-uracil))-ethyl vinyl adipate]-D-glucose (5e)

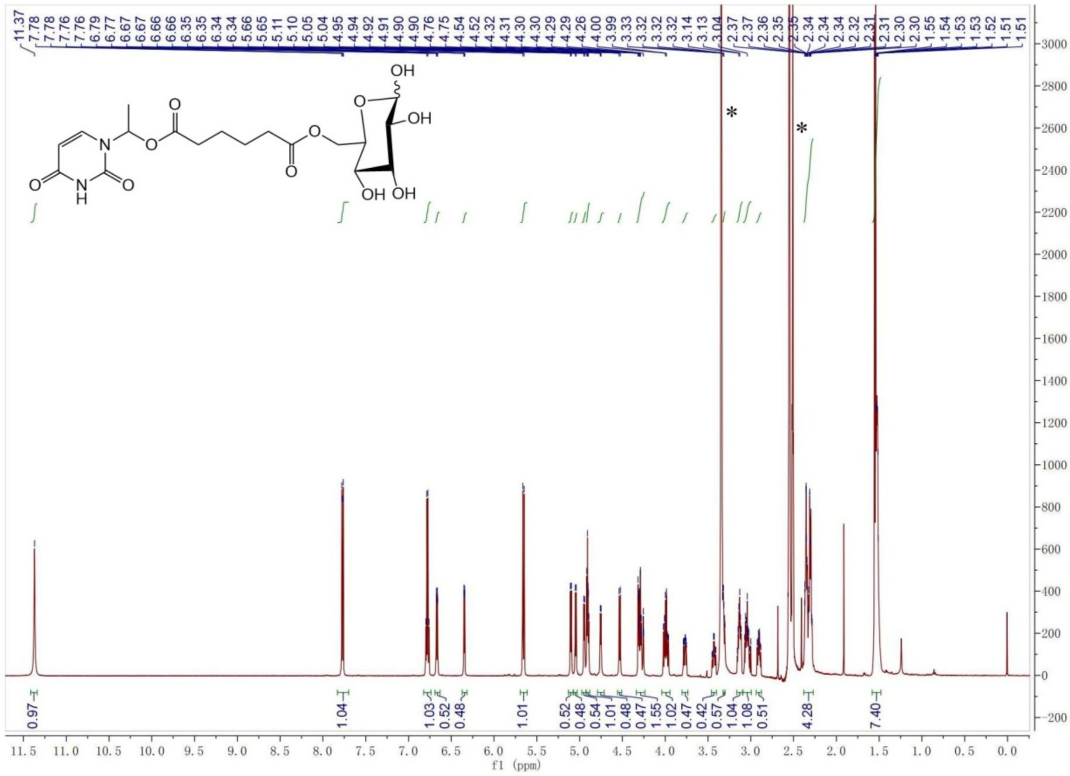

6’-O-[1-(1-uracil))-ethyl vinyl adipate]-D-mannose (5f)

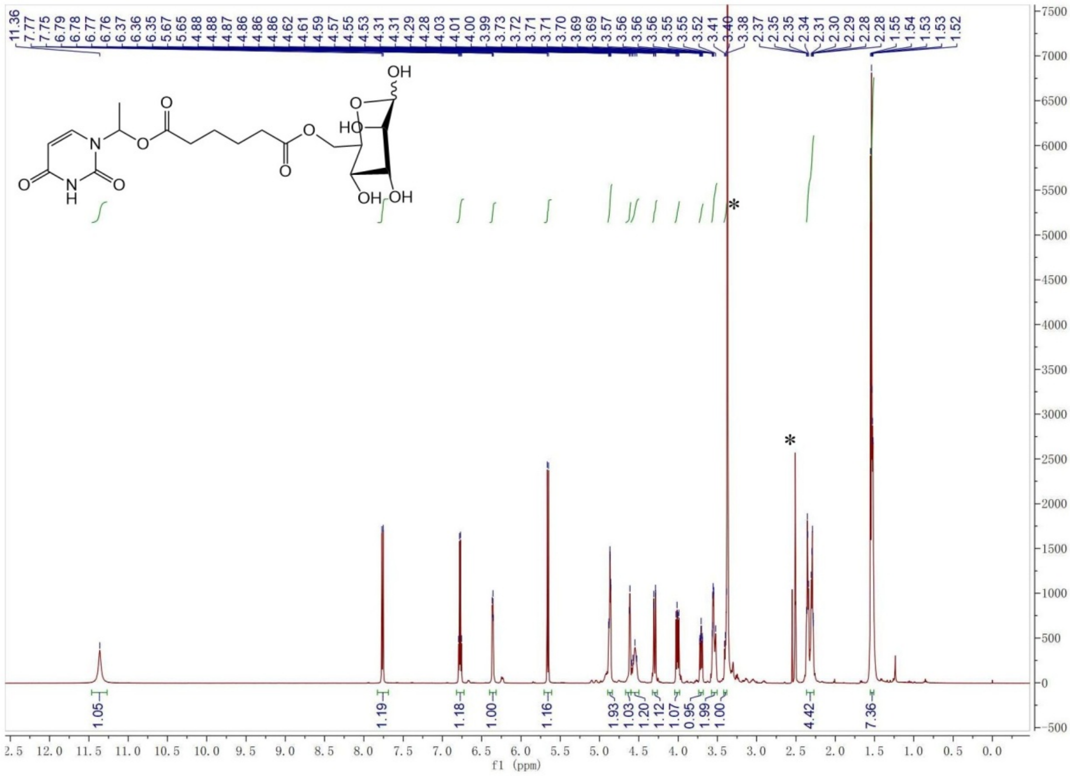

6’-O-[1-(1-uracil))-ethyl vinyl adipate]-sucrose (5g)

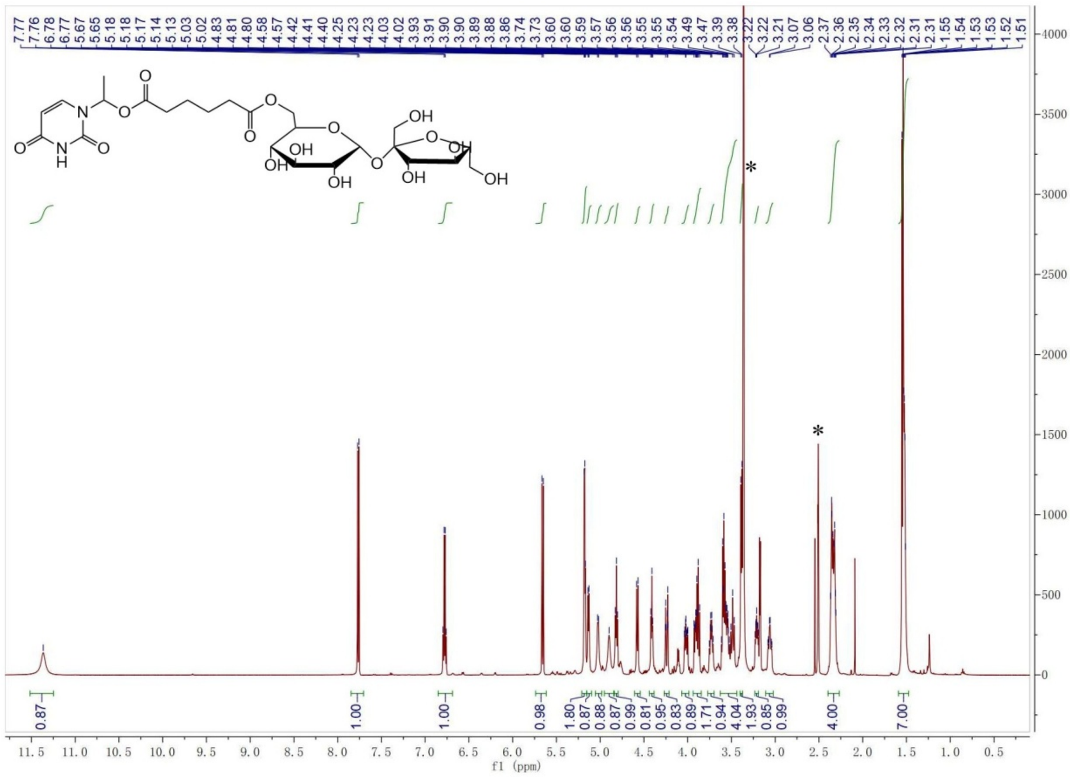

6’-O-[1-(1-uracil))-ethyl vinyl adipate]-maltose (5h)

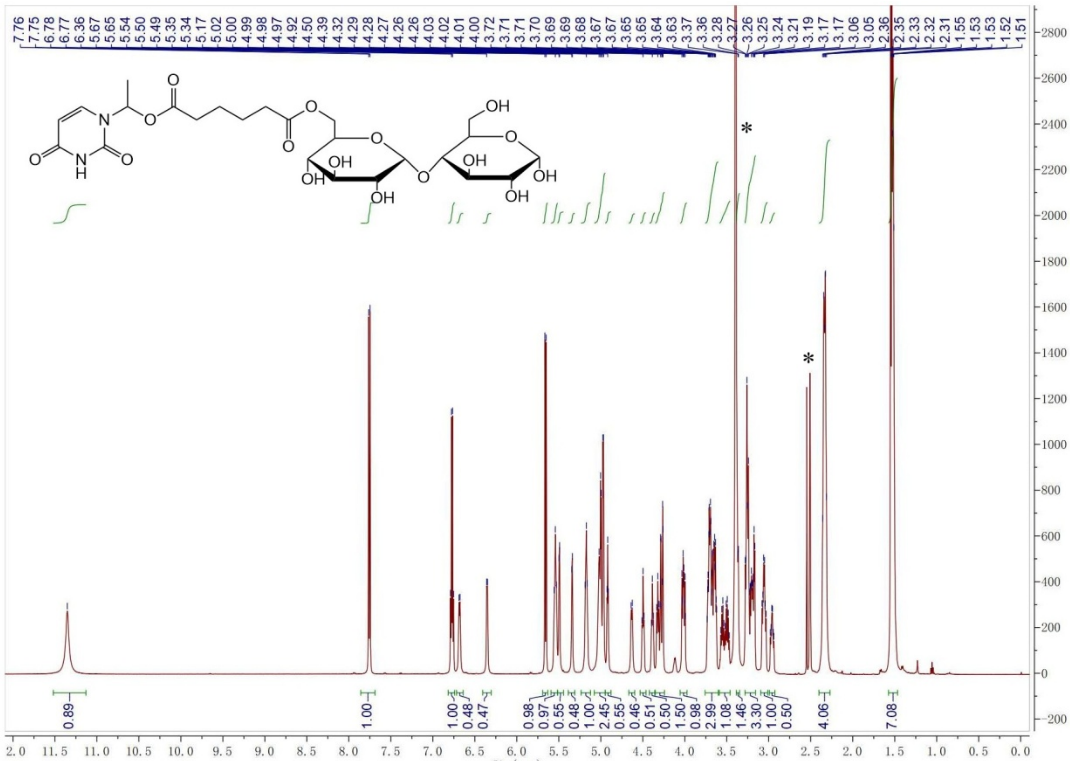

6’-O-[1-(1-thymine)-ethyl vinyl adipate]-D-glucose (5i)

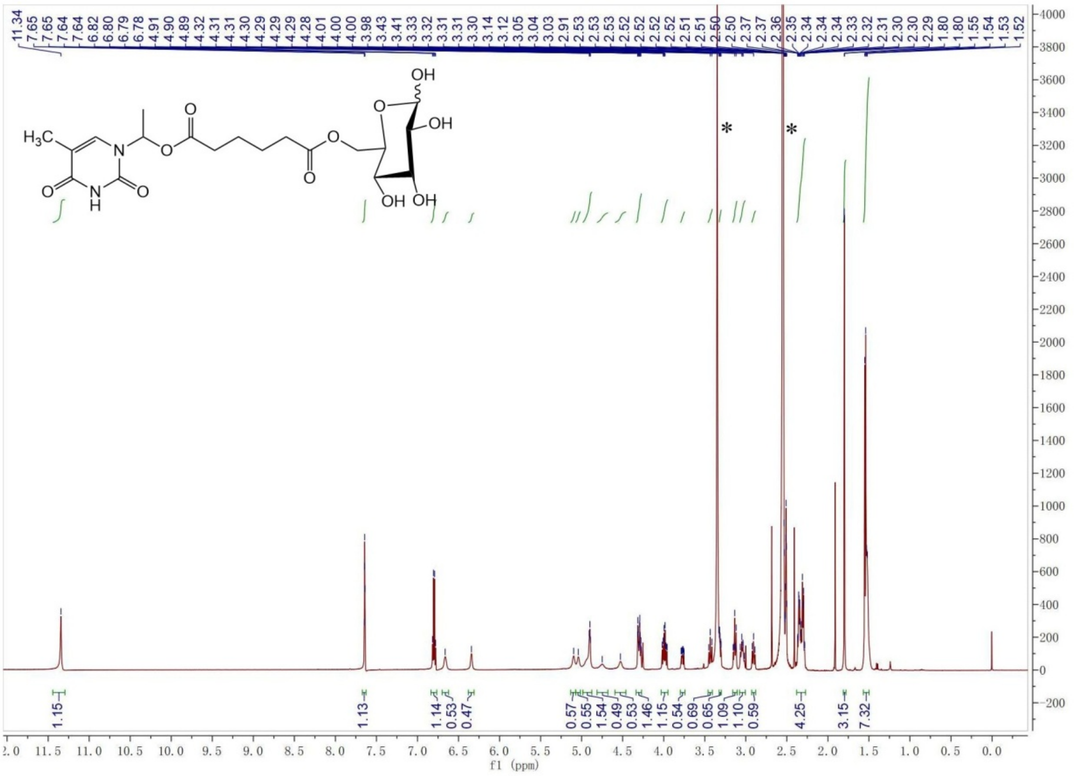

6’-O-[1-(1-thymine)-ethyl vinyl adipate]-D-mannose (5j)

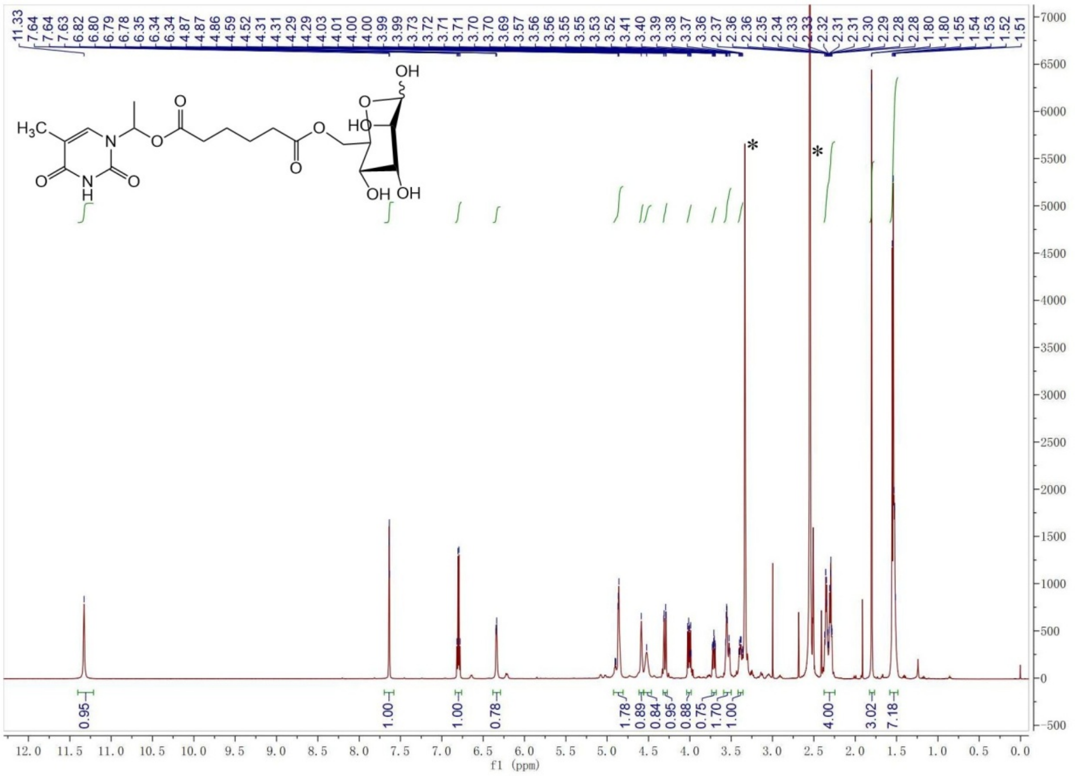

6’-O-[1-(1-thymine)-ethyl vinyl adipate]- sucrose (5k)

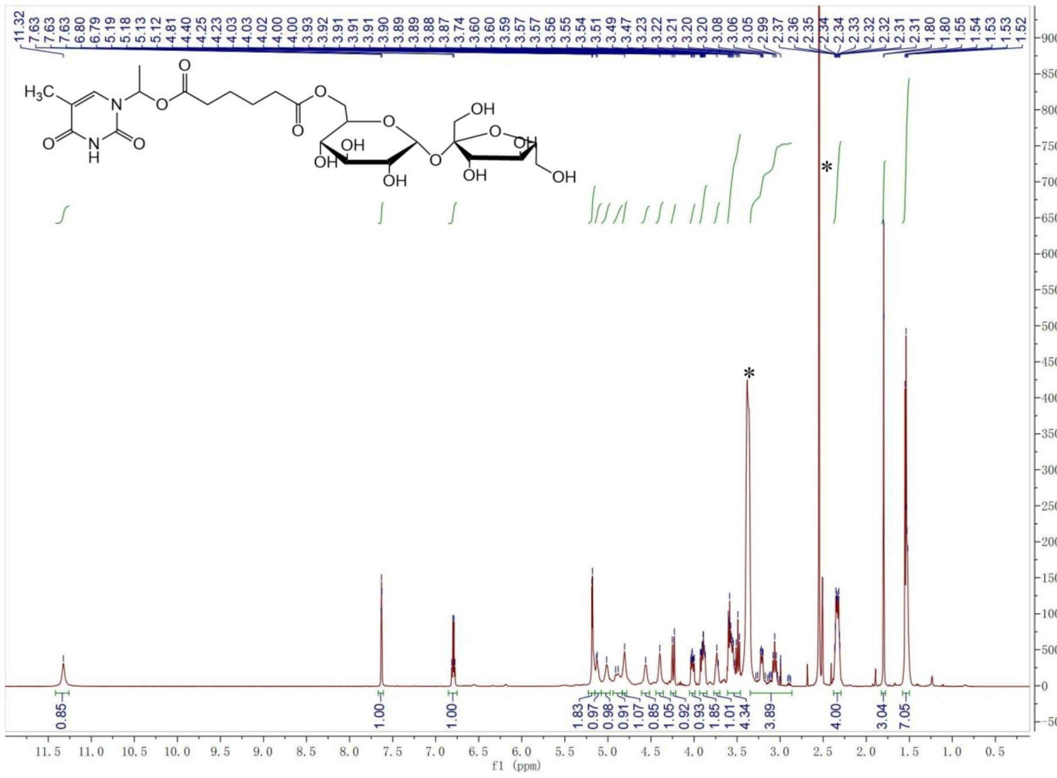

6’-O-[1-(1-thymine)-ethyl vinyl adipate]-maltose (5l)

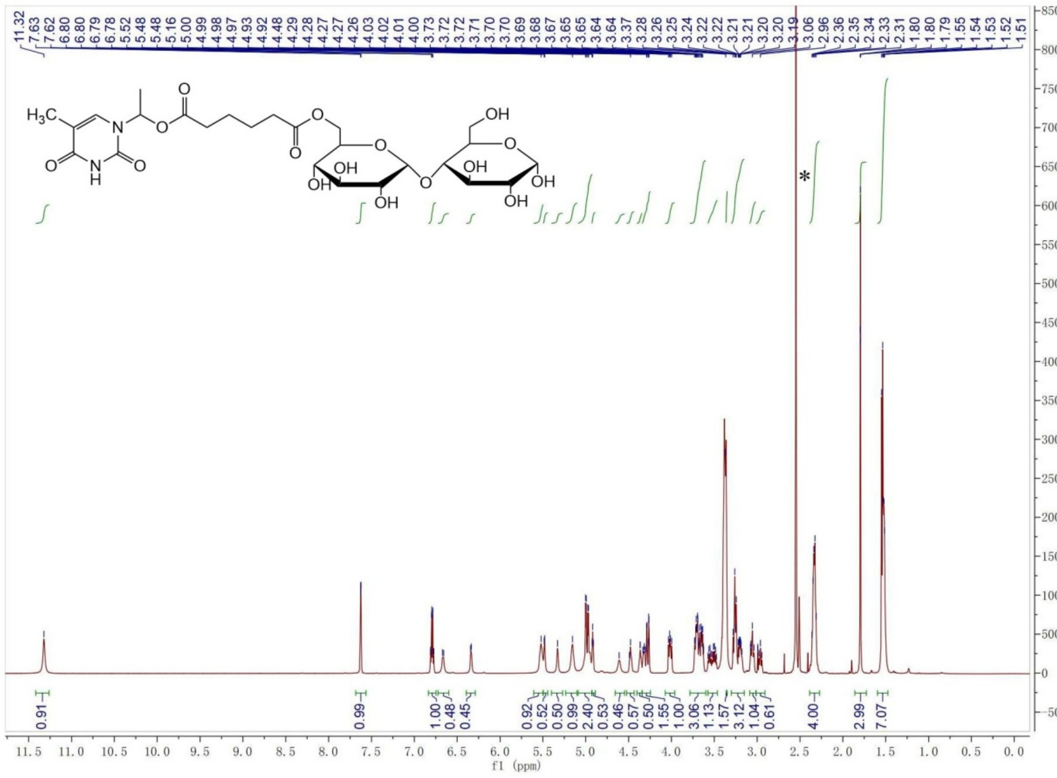

6’-O-[1-(1-(5-Fluououracil))-ethyl vinyl adipate]-D-glucose (5a)

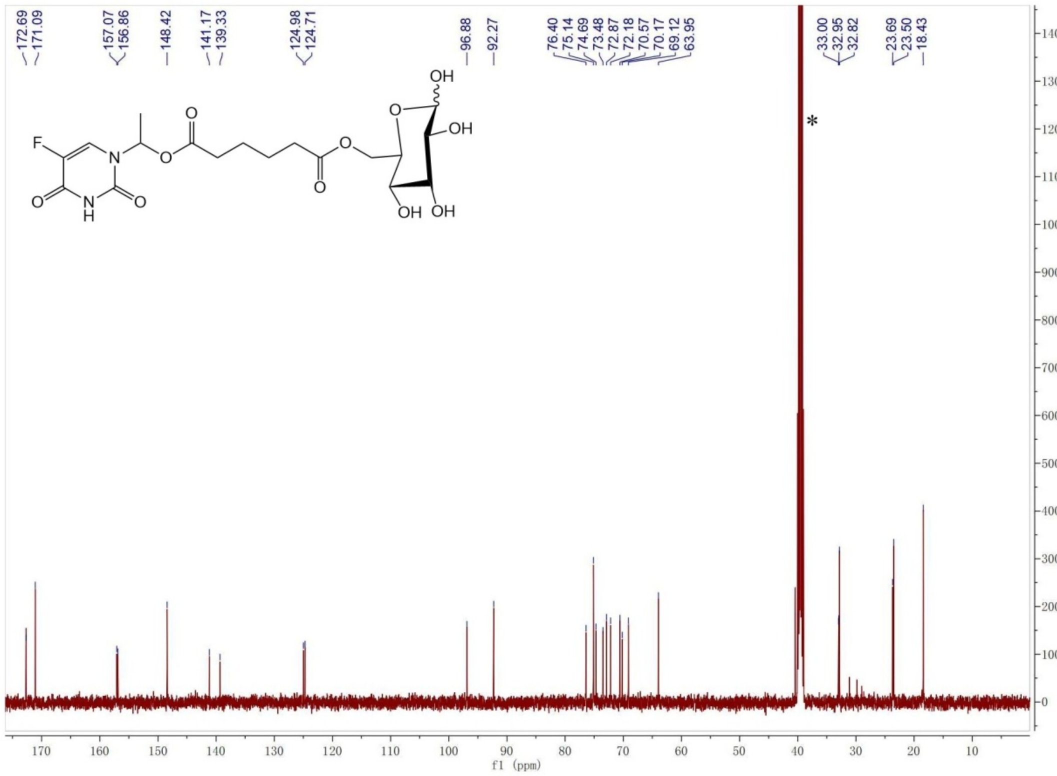

6’-O-[1-(1-(5-Fluououracil))-ethyl vinyl adipate]-D-mannose (5b)

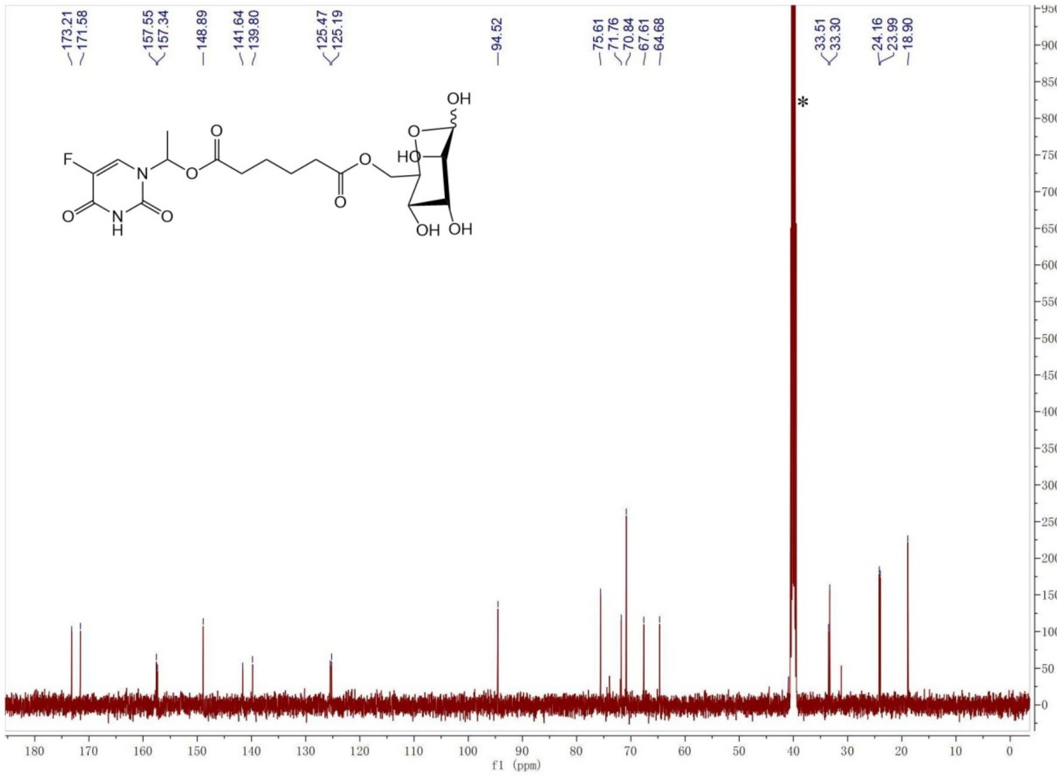

6’-O-[1-(1-(5-Fluououracil))-ethyl vinyl adipate]-sucrose (5c)

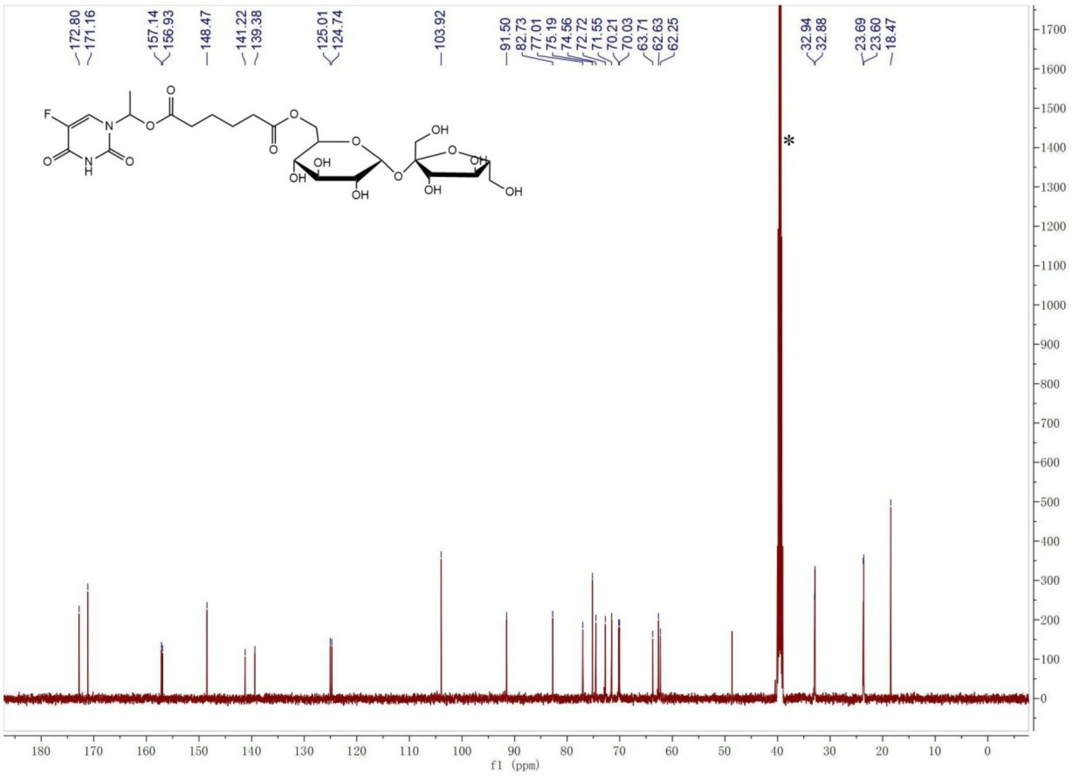

6’-O-[1-(1-uracil))-ethyl vinyl adipate]-D-glucose (5e)

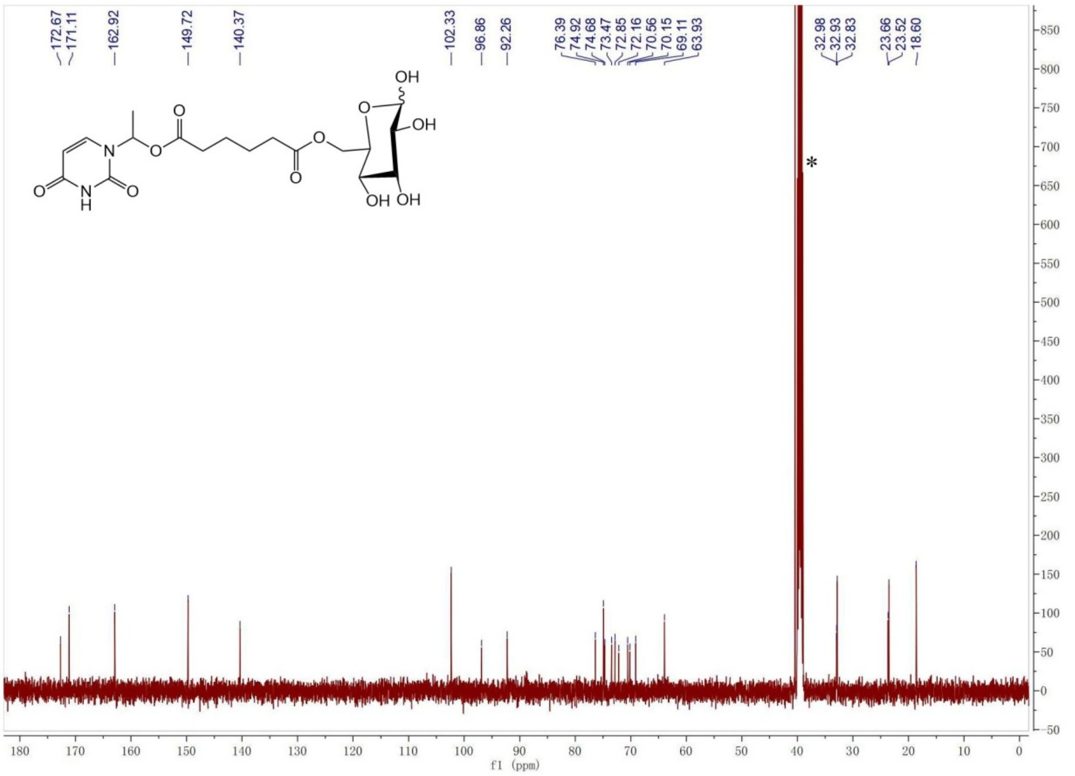

6’-O-[1-(1-uracil))-ethyl vinyl adipate]-D-mannose (5f)

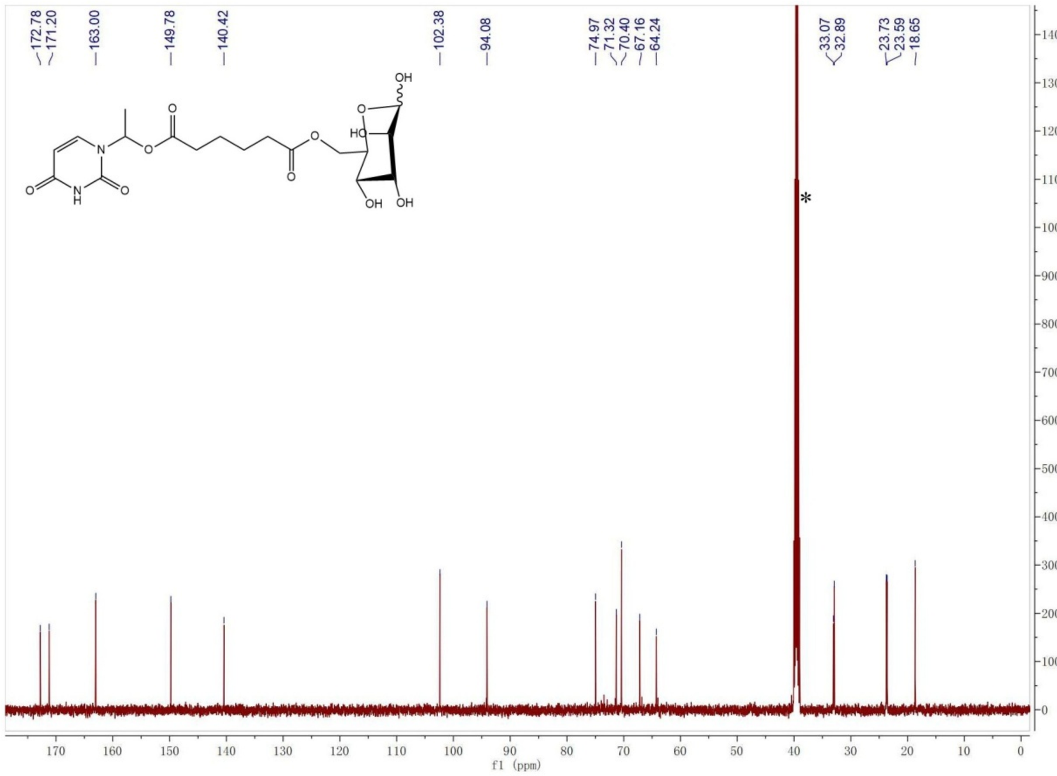

6’-O-[1-(1-uracil))-ethyl vinyl adipate]-sucrose (5g)

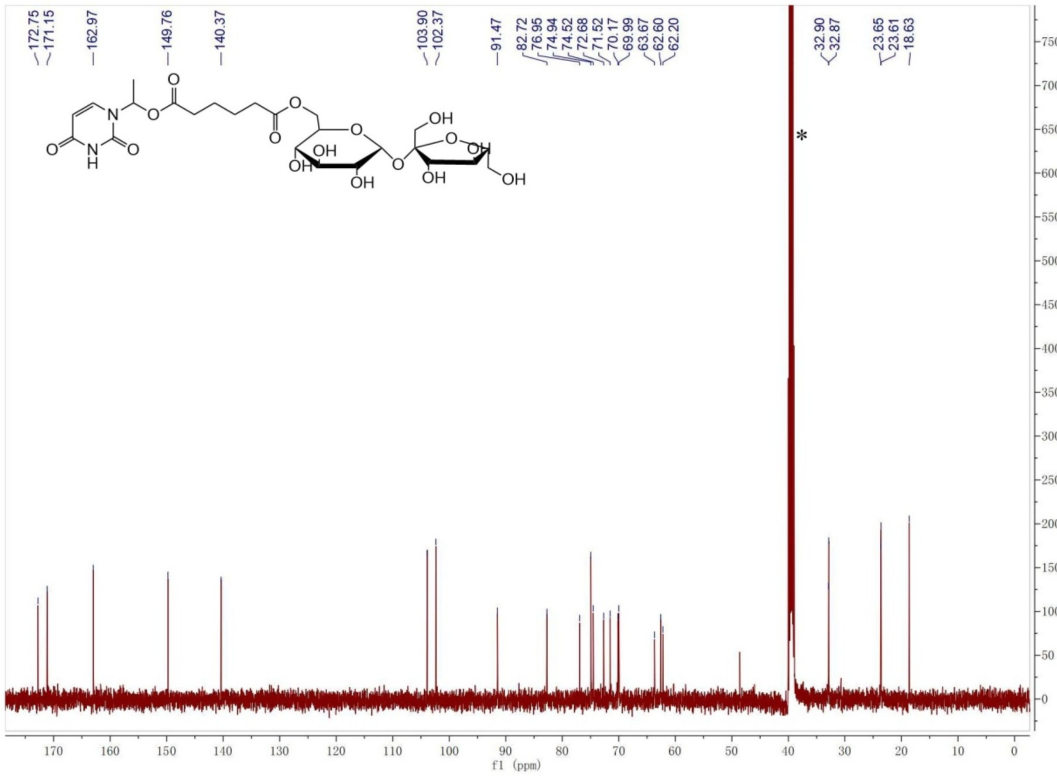

6’-O-[1-(1-uracil))-ethyl vinyl adipate]-maltose (5h)

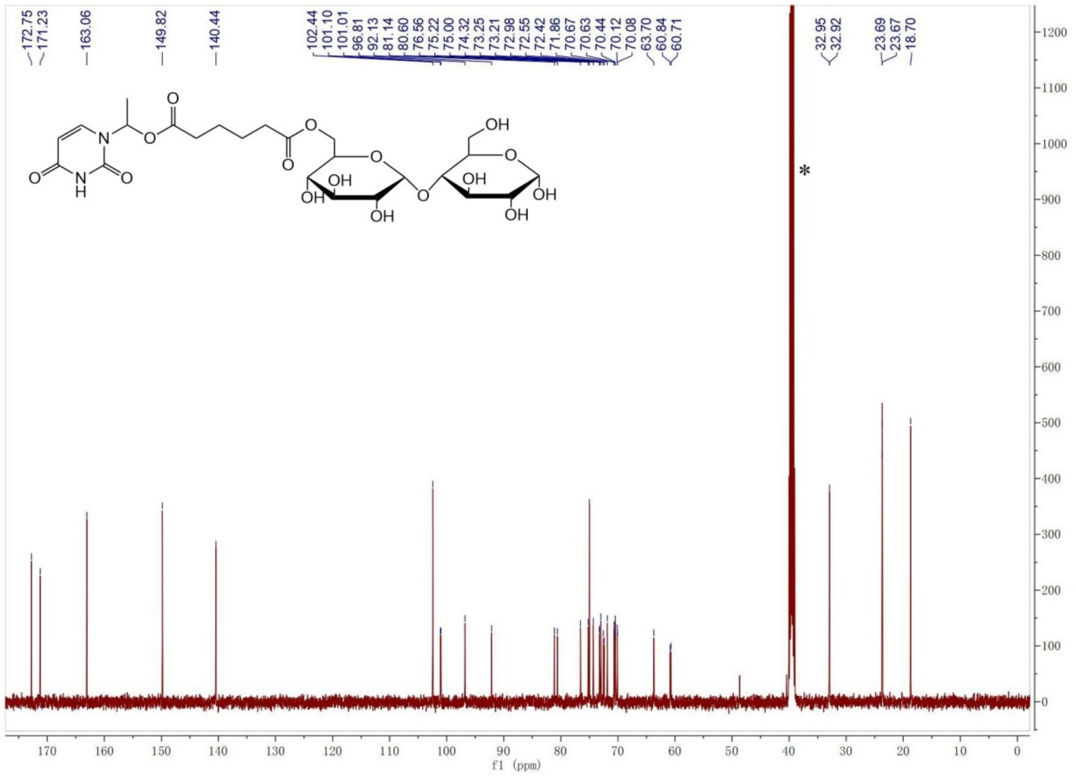

6’-O-[1-(1-thymine)-ethyl vinyl adipate]-D-glucose (5i)

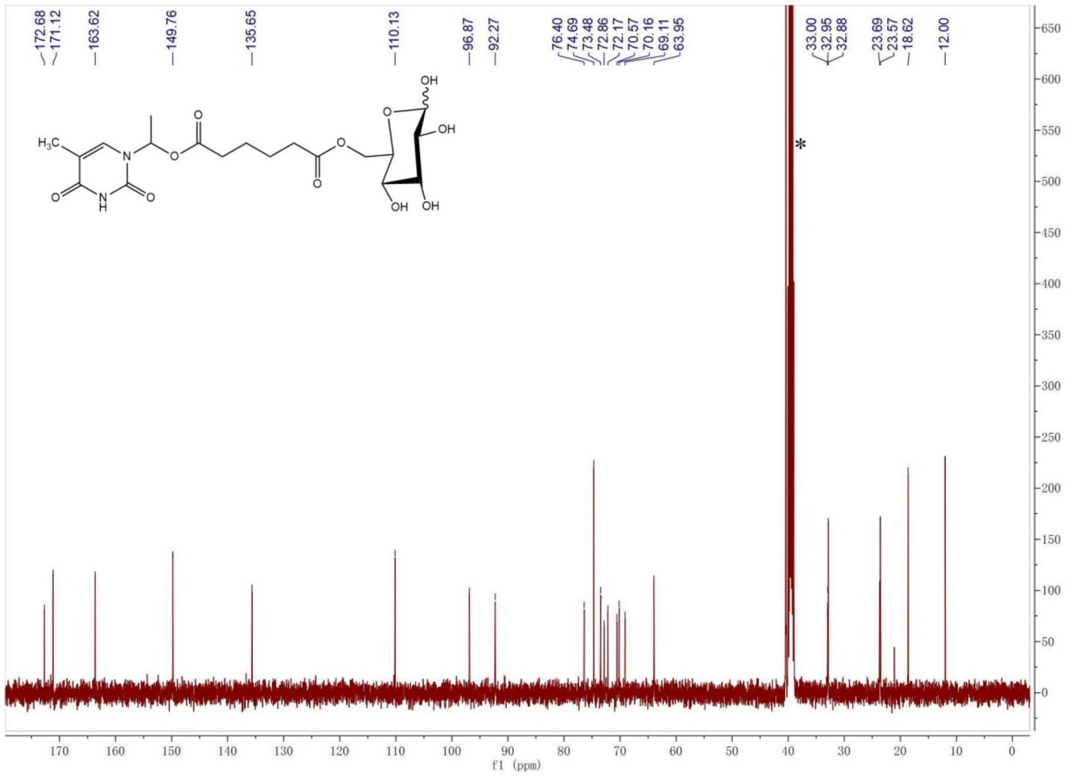

6’-O-[1-(1-thymine)-ethyl vinyl adipate]-D-mannose (5j)

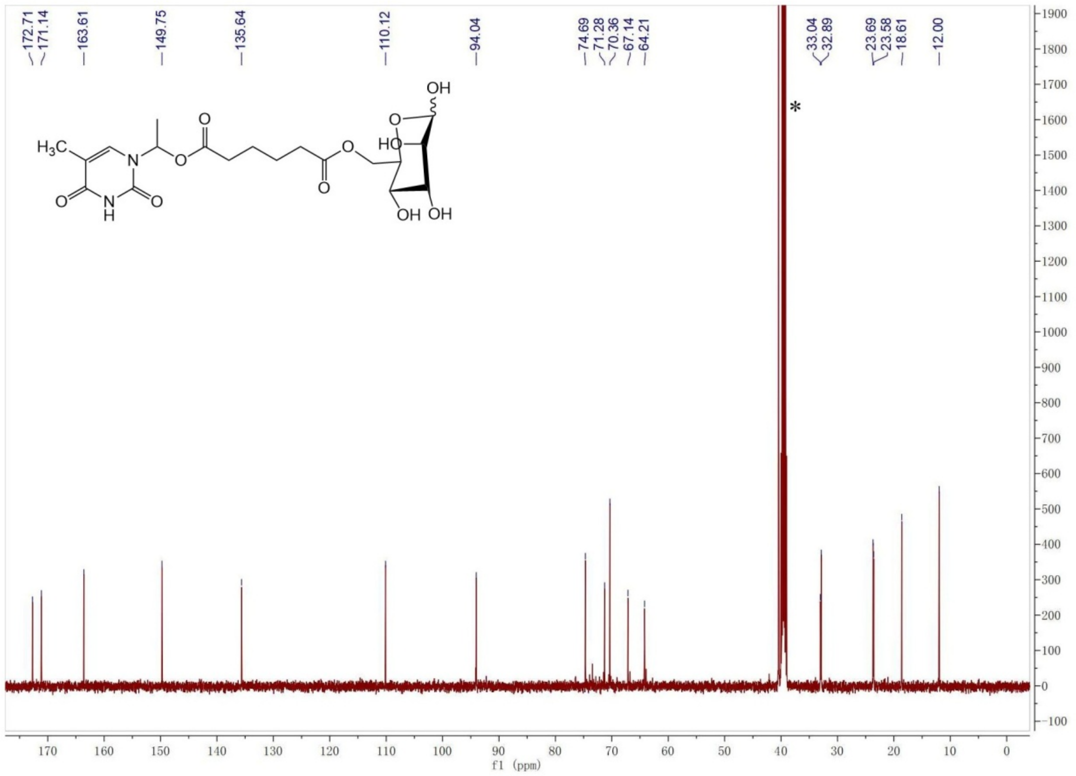

6’-O-[1-(1-thymine)-ethyl vinyl adipate]-sucrose (5k)

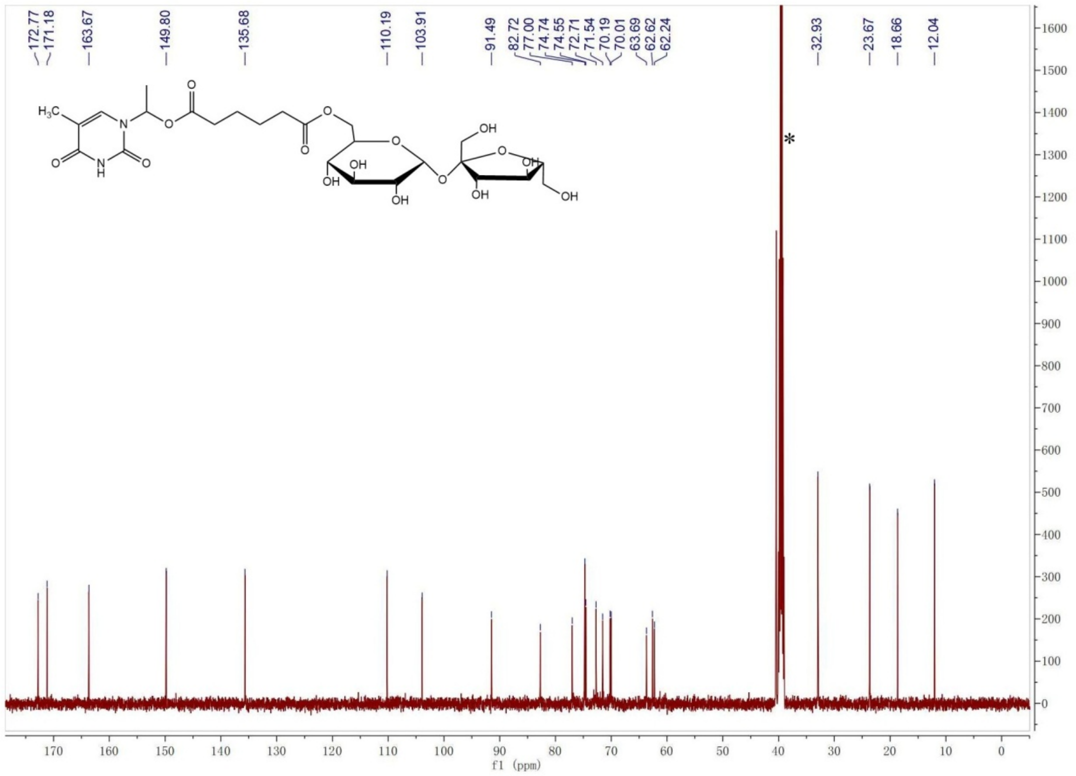

6′-O-[1-(1-(5-Fluorouracil))-ethyl vinyl adipate]-D-glucose (5a)

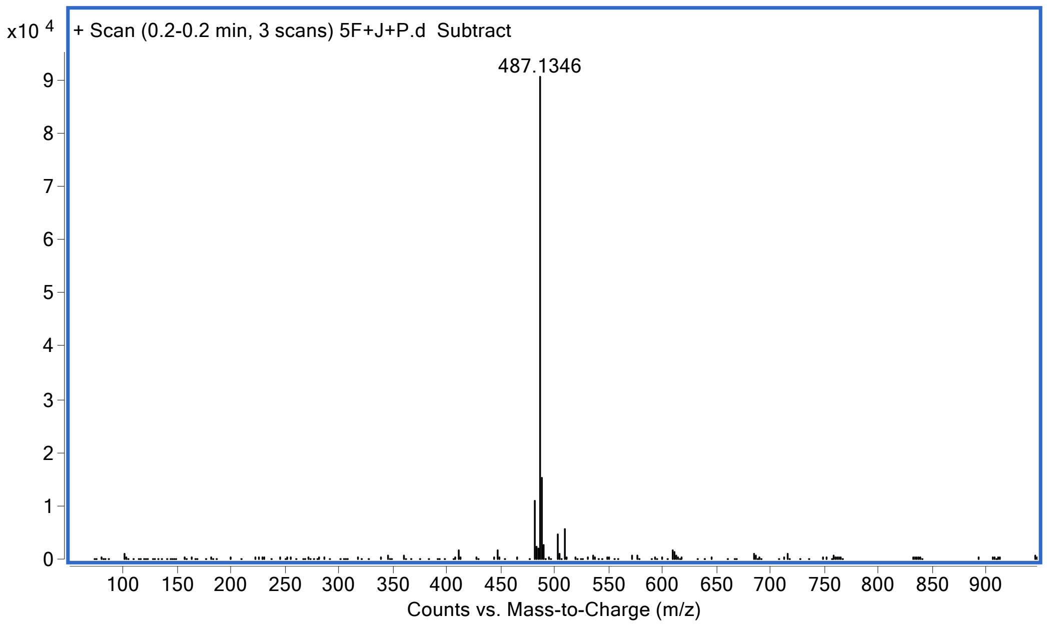

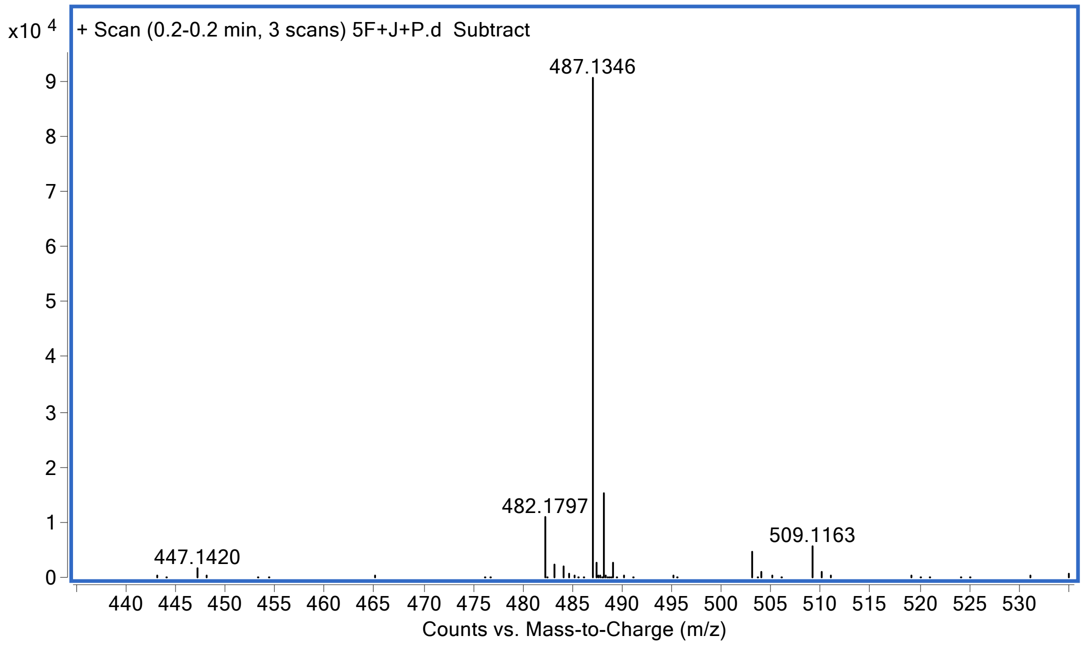

6′-O-[1-(1-(5-Fluouoruracil))-ethyl vinyl adipate]-D-mannose (5b)

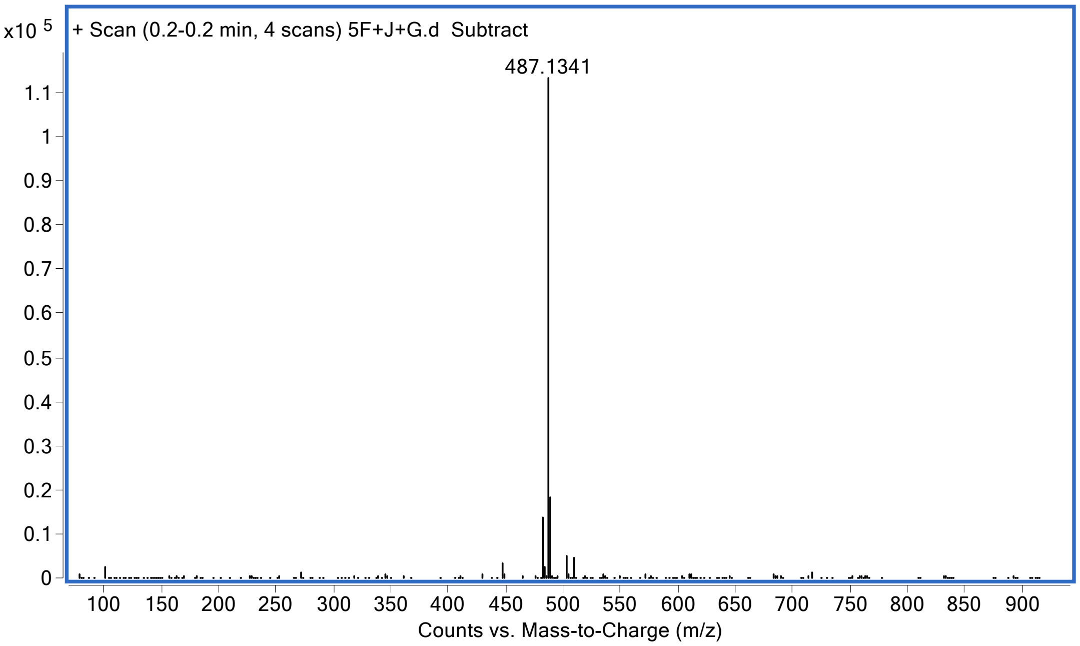
x10 5
+ Scan (0.2-0.2 min, 4 scans) 5F+J+G.d Subtract
487.1341
1.1
1
0.9
0.8
0.7
0.6
0.5
0.4
0.3
0.2
0.1
0
100 150 200 250 300 350 400 450 500 550 600 650 700 750 800 850 900
Counts vs. Mass-to-Charge (m/z)

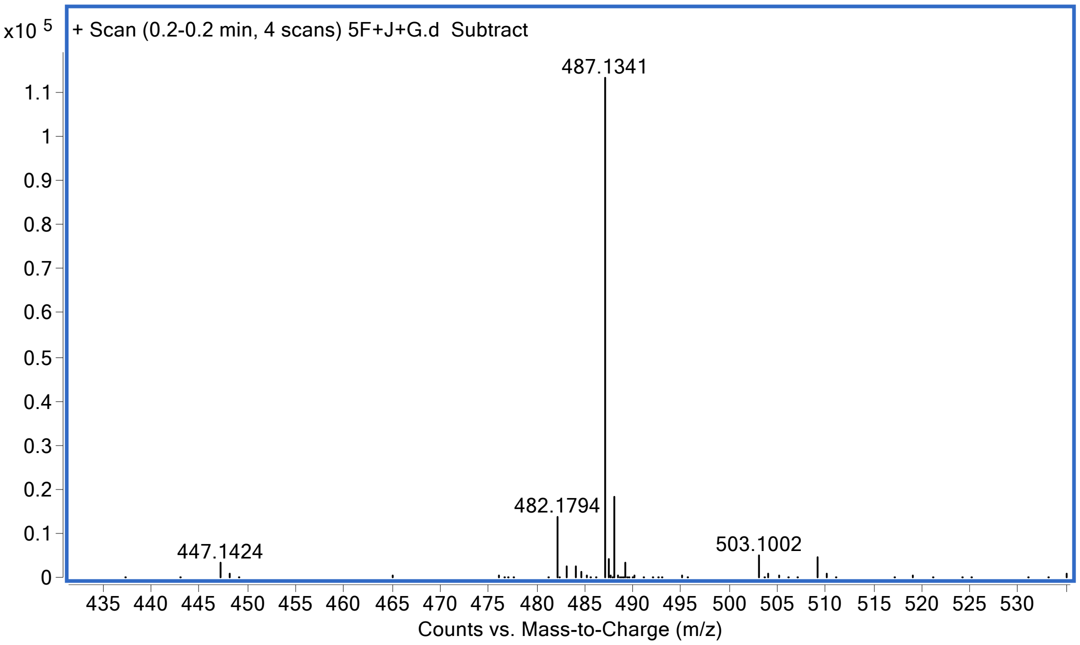
x10 5
+ Scan (0.2-0.2 min, 4 scans) 5F+J+G.d Subtract
487.1341
482.1794
447.1424
503.1002
1.1
1
0.9
0.8
0.7
0.6
0.5
0.4
0.3
0.2
0.1
0
435 440 445 450 455 460 465 470 475 480 485 490 495 500 505 510 515 520 525 530
Counts vs. Mass-to-Charge (m/z)
